# The protein phosphatase OsPP1a dephosphorylates and stabilizes CatC to scavenge excess H_2_O_2_ and enhance salt tolerance in rice

**DOI:** 10.1016/j.xplc.2026.101824

**Published:** 2026-03-16

**Authors:** Yan Wang, YuTing Yi, ZeLin Xu, Ye Tian, DeMing Mao, ZhenDie Luo, ZhengKun Zhou, Sheng- Nan Hu, YanNing Tan, XinHui Zhao, Lei Yang, DongYing Tang, YuanZhu Yang, WenBang Tang, Cong Liu, XuanMing Liu, JianZhong Lin

**Affiliations:** 1 Yuelushan Laboratory, Hunan Province Key Laboratory of Plant Functional Genomics and Developmental Regulation, Hunan Research Center of the Basic Discipline for Cell Signaling, State Key Laboratory of Chemo/Biosensing and Chemometrics, Longping Agricultural College, College of Biology, Hunan University, Changsha 410082, China; 2 College of Bioscience and Biotechnology, Hunan Agricultural University, Changsha 410128, China; 3 State Key Laboratory of Hybrid Rice, Hunan Hybrid Rice Research Center, Hunan Academy of Agricultural Sciences, Changsha 410125, China; 4 Key Laboratory of Southern Rice Innovation & Improvement, Ministry of Agriculture and Rural Affairs/Hunan Engineering Laboratory of Disease and Pest Resistant Rice Breeding, Yuan Longping High-Tech Agriculture Co., Ltd., Changsha 410001, China; 5 National Center of Technology Innovation for Saline-Alkali Tolerant Rice, Changsha 410125, China; 6 Greater Bay Area Institute for Innovation, Hunan University, Guangzhou 511300, China

**Keywords:** rice, OsPP1a, CatC stability, APIP6, H_2_O_2_ homeostasis, salt tolerance

## Abstract

Catalase (CAT) plays a central role in maintaining H_2_O_2_ homeostasis during stress responses; however, how phosphatase-mediated dephosphorylation modulates CAT activity and stability remains unclear. Here, we identify the protein phosphatase OsPP1a as a positive regulator of salt tolerance in rice (*Oryza sativa*). OsPP1a directly dephosphorylates CatC at Thr-292 in the peroxisome, thereby enhancing its stability and enzymatic activity by inhibiting ubiquitination and degradation mediated by the E3 ubiquitin ligase APIP6. Consistently, *OsPP1a*-overexpressing lines exhibit enhanced tolerance to salt and oxidative stress, accompanied by reduced phospho-threonine levels of CAT proteins. Phosphatase activity and seminal root growth assays further demonstrate that OsPP1a acts as a key regulator balancing salt tolerance and growth in rice. Importantly, overexpression of *OsPP1a* markedly alleviates salt-induced grain yield loss. Together, these findings elucidate a mechanism by which phosphatase-mediated dephosphorylation activates and stabilizes CAT, and provide a potential strategy for breeding salt-tolerant rice varieties.

## Teaser

This study reports that the protein phosphatase OsPP1a dephosphorylates catalase CatC to enhance its stability and activity by inhibiting ubiquitination and degradation, thereby promoting H_2_O_2_ scavenging and improving salt tolerance in rice.

## Introduction

Salinity is a major ecological constraint that impairs crop growth and productivity, thereby hindering sustainable agricultural development and threatening global food security. With the ongoing expansion of soil salinization and the increasing global demand for food, the development of saline–alkali land, the identification of salt-tolerant genes, and the breeding of salt-tolerant rice (*Oryza sativa*) varieties have become increasingly urgent and essential (Liu et al., 2024). Under salt stress, in addition to osmotic stress and sodium (Na^+^) toxicity, the excessive accumulation of reactive oxygen species (ROS) in cellular compartments such as mitochondria, chloroplasts, the apoplast, and peroxisomes acts as a secondary stressor, further compromising plant performance. While low steady-state levels of ROS function as signaling molecules that regulate plant growth, development, and stress responses (Mittler, 2017), excessive ROS accumulation is detrimental to cellular activities (Suzuki et al., 2012). Thus, maintaining a balance between ROS production and detoxification is critical for normal cellular function (Szechyńska-Hebda et al., 2022). Plants have evolved efficient enzymatic and non-enzymatic detoxification systems to maintain ROS homeostasis. Enzymatic ROS-scavenging systems include catalase (CAT), superoxide dismutase, ascorbate peroxidase, and glutathione peroxidase (Apel and Hirt, 2004; Mittler et al., 2004). Non-enzymatic antioxidants comprise major cellular redox buffers such as glutathione and ascorbate, as well as secondary metabolites including flavonoids, alkaloids, tocopherols, and carotenoids (Wang et al., 2023a). Collectively, these scavenging enzymes and antioxidant metabolites constitute a highly efficient system for ROS detoxification, thereby maintaining redox homeostasis.

Hydrogen peroxide (H_2_O_2_), a key type of ROS, plays a dual role in plants (Waszczak et al., 2018). At low concentrations, H_2_O_2_ functions as a signaling molecule that orchestrates plant growth, development, and stress responses (Miller et al., 2010; Evans et al., 2016). For example, low levels of H_2_O_2_ activate its sensor, HYDROGEN PEROXIDE-INDUCED Ca^2+^ INCREASES (HPCA1), to regulate stomatal closure as well as growth and development in Arabidopsis (*Arabidopsis thaliana*) (Wu et al., 2020). In contrast, high levels of H_2_O_2_ cause DNA damage, leading to crosslinks, base modifications, deletions, and genomic instability (Chandrakar et al., 2017). Excessive H_2_O_2_ accumulation under alkaline stress significantly inhibits crop growth and yield, whereas the disinhibition of the H_2_O_2_ exporter PIP2;1 by the Gγ subunit Alkaline tolerance 1 (AT1) maintains H_2_O_2_ homeostasis and enhances alkaline tolerance (Zhang et al., 2023). CAT is a key antioxidant enzyme that decomposes excess H_2_O_2_ generated under stress conditions, thereby protecting plant cells. Multiple CAT isoforms exist in plants and exhibit diverse physiological functions. In Arabidopsis, three CAT isoforms—CAT1, CAT2, and CAT3—have been identified. Among these, CAT2 accounts for the majority of CAT activity, whereas CAT3 displays relatively low catalytic activity but high transnitrosylase activity; CAT1 shows limited expression and functionality (Chen et al., 2020). Similarly, rice possesses three CAT isoforms—CatA, CatB, and CatC—corresponding to Arabidopsis CAT3, CAT1, and CAT2, respectively (Joo et al., 2014; Chen et al., 2020). Although CatA lacks catalase activity, it participates in nitric oxide signaling through its transnitrosylase activity (Chen et al., 2020). CatB contributes to high-temperature tolerance and is involved in immune responses (Liu et al., 2020; Gao et al., 2021), whereas CatC enhances salt tolerance, albeit with increased susceptibility to the rice blast pathogen *Magnaporthe oryzae* (Zhou et al., 2018; You et al., 2022). Collectively, these findings underscore the critical role of CAT in maintaining H_2_O_2_ homeostasis.

CAT activity is closely associated with its oligomeric state and is regulated by specific interacting proteins as well as reversible protein phosphorylation (Li et al., 2015). Monomeric CAT exhibits little or no activity, whereas its homotetramer displays markedly enhanced catalytic activity (Liu et al., 2019; Wang et al., 2023b). The chaperone NO CATALASE ACTIVITY1 (NCA1) and the WD40 protein TaWD40-4B.1 promote CAT tetramer assembly and activity in Arabidopsis and wheat (*Triticum aestivum*), respectively (Li et al., 2015; Tian et al., 2023). Phosphorylation plays a key role in regulating CAT activity across species. In humans (Homo sapiens), protein kinase Cδ (PKCδ) phosphorylates CAT at Ser-167, promoting its tetramerization and activity (Rafikov et al., 2014). In plants, calcium-dependent protein kinase 8 (CPK8) phosphorylates CAT3 at Ser-261, enhancing its activity and enhancing drought tolerance in Arabidopsis (Zou et al., 2015). In rice, SALT TOLERANCE RECEPTOR-LIKE CYTOPLASMIC KINASE 1 (STRK1) phosphorylates CatC at Tyr-210, thereby promoting its activity and enhancing salt tolerance (Zhou et al., 2018). This modification is dependent on STRK1 S-acylation mediated by the DHHC-type zinc-finger protein DHHC09 (Tian et al., 2024). Additionally, BRI1-ASSOCIATED RECEPTOR KINASE 1 (BAK1) phosphorylates and activates CAT3, reducing H_2_O_2_ levels and inhibiting plant growth in Arabidopsis (Zhang et al., 2020c). In rice, a calcium-dependent protein kinase (OsCPK12) phosphorylates CatC at Ser-11, thereby modulating H_2_O_2_ homeostasis and enhancing oxidative stress tolerance (Wang et al., 2023a). In contrast, protein phosphatases generally act as negative regulators of CAT activity. For example, PHOSPHATASE OF CATALASE 1 (PC1) dephosphorylates CatC at Ser-9, inhibiting its tetramerization and negatively regulating salt tolerance in rice (Liu et al., 2023). Similarly, protein phosphatase 2C1 (PP2C1) dephosphorylates CAT at Ser-112, reducing its activity and stress tolerance in cassava (*Manihot esculenta*) (Bai et al., 2024). Notably, dephosphorylation of CatC at Ser-18 promotes its tetramerization and enhances salt tolerance in rice; however, the responsible phosphatase has not been identified (Wang et al., 2023b). In addition to post-translational modification, CAT activity is closely linked to protein stability, with degradation mediated by the ubiquitin–26S proteasome pathway. The E3 ubiquitin ligases AvrPiz-t Interacting Protein 6 (APIP6) and CAT2 Interacting RING Protein 1 (CIRP1) ubiquitinate CAT, promoting its degradation via the 26S proteasome and enhancing blast resistance in rice and drought tolerance in Arabidopsis, respectively (You et al., 2022; Yang et al., 2025). Interestingly, the *M. oryzae* effector AvrPiz-t structurally mimics the susceptibility protein RESISTANCE OF RICE TO DISEASES 1 (ROD1), and both proteins can activate CatB to eliminate ROS while also being ubiquitinated by APIP6 (Park et al., 2012; Gao et al., 2021). These findings indicate that the ubiquitin–proteasome system plays a critical role in CAT-mediated ROS homeostasis and stress responses. Although CAT is generally considered to be activated by kinases and inactivated by phosphatases, the molecular mechanisms underlying phosphatase-mediated activation of CAT, as well as the regulation of its stability by phosphorylation, remain largely unclear.

The plant protein phosphatase family comprises phosphoprotein phosphatases (further subdivided into PP1, PP2A, PP2B, and PP4–PP7), metal-dependent protein phosphatases/protein phosphatase 2C (PP2C), protein tyrosine phosphatases, and aspartate-dependent phosphatases (Shi, 2009; Bheri et al., 2021). Among these, PP1 plays a key role in regulating plant growth, development, and stress responses (Wang et al., 2022). In Arabidopsis, nine PP1 members have been identified, designated TOPP1–TOPP9 (Lin et al., 1998), whereas rice contains five PP1 members, named OsPP1a–OsPP1e (Ogawa et al., 2011). For example, Protein Phosphatase 1a (OsPP1a) and TdPP1a act as positive regulators of salt stress tolerance by promoting ROS scavenging in rice and wheat, respectively (Liao et al., 2016; Bradai et al., 2018), although their underlying mechanisms remain unclear. Here, we demonstrate that OsPP1a specifically dephosphorylates CatC at Thr-292, thereby inhibiting its APIP6-mediated ubiquitination and degradation. This mechanism contributes to the maintenance of H_2_O_2_ homeostasis and enhances salt tolerance in rice. Moreover, overexpression of *OsPP1a* not only improves seedling growth but also markedly reduces grain yield loss under salt stress.

## Results

### The protein phosphatase OsPP1a enhances tolerance to salt and oxidative stresses in rice

Although CAT stability has been reported to be mediated by the ubiquitin–26S proteasome pathway (You et al., 2022), the underlying regulatory mechanisms remain unclear. To identify new regulators of CAT stability, we performed immunoprecipitation–mass spectrometry analysis of the CAT protein complex in rice. The CatC protein complex was immunoprecipitated using anti-FLAG beads from protein extracts of *FLAG-CatC-*overexpressing rice seedlings, followed by mass spectrometric identification of associated proteins. As a result, the protein phosphatase OsPP1a was identified as a component of the CatC complex (Supplemental Table 1). Phylogenetic analysis classified OsPP1a within the PP1 family and revealed high sequence similarity (up to 85%) to the phosphatase AtTOPP4 (Supplemental Figures 1A and 1B), which has been implicated in salt tolerance in Arabidopsis (Shen, 2017). Notably, OsPP1a has been previously reported to act as a positive regulator of salt stress by promoting ROS scavenging in rice (Liao et al., 2016). Therefore, we hypothesized that OsPP1a may regulate CAT stability or function, particularly under salt stress. Subcellular localization analysis showed that fluorescence signals from both N-terminal green fluorescent protein (GFP)-OsPP1a and C-terminal OsPP1a-GFP fusion proteins strongly overlapped with those of the peroxisomal marker cyan fluorescent protein (CFP)-PTS1 in rice protoplasts, indicating that OsPP1a localizes to the peroxisome (Supplemental Figure 2A). Furthermore, tissue-specific expression analysis revealed that *OsPP1a* is predominantly expressed in leaves and is induced by sodium chloride (NaCl), polyethylene glycol (PEG), alkali, and H_2_O_2_ treatments (Supplemental Figures 2B–2K), suggesting that OsPP1a is involved in responses to salt and oxidative stresses in rice.

To elucidate the function of OsPP1a in salt and oxidative stress responses, we generated *OsPP1a*-overexpressing and knockout lines (Supplemental Figures 3A and 3B). Given that OsPP1a shares more than 74% sequence similarity with OsPP1b and OsPP1c (Supplemental Figure 1B), functional redundancy among these proteins was anticipated. Thus, we constructed *ospp1a ospp1b* and *ospp1a ospp1c* double mutants in the *ospp1a-23* background (Supplemental Figures 3C and 3D). Notably, the *ospp1a ospp1c* double mutants exhibited severe dwarfism and sterility (Supplemental Figure 3E). Therefore, for subsequent phenotypic analyses under salt stress (140 mM NaCl), we used *ospp1a(−/−) ospp1c(+/−)* heterozygotes, as well as *ospp1a* and *ospp1a ospp1b* homozygous mutants. Compared with wild-type (WT) plants, all these mutant lines showed increased sensitivity to salt stress (Supplemental Figures 3F and 3G). In particular, *ospp1a ospp1b* double mutants displayed greater sensitivity and lower survival rates than *ospp1a* single mutants, whereas no significant difference was observed between *ospp1a* mutants and *ospp1a(−/−) ospp1c(+/−)* heterozygotes. These results indicate that OsPP1a and OsPP1b function synergistically to enhance salt tolerance in rice, whereas the contribution of OsPP1c is relatively minor or not evident under the tested conditions. Therefore, subsequent analyses focused on the biological function of OsPP1a.

**Figure 1:**
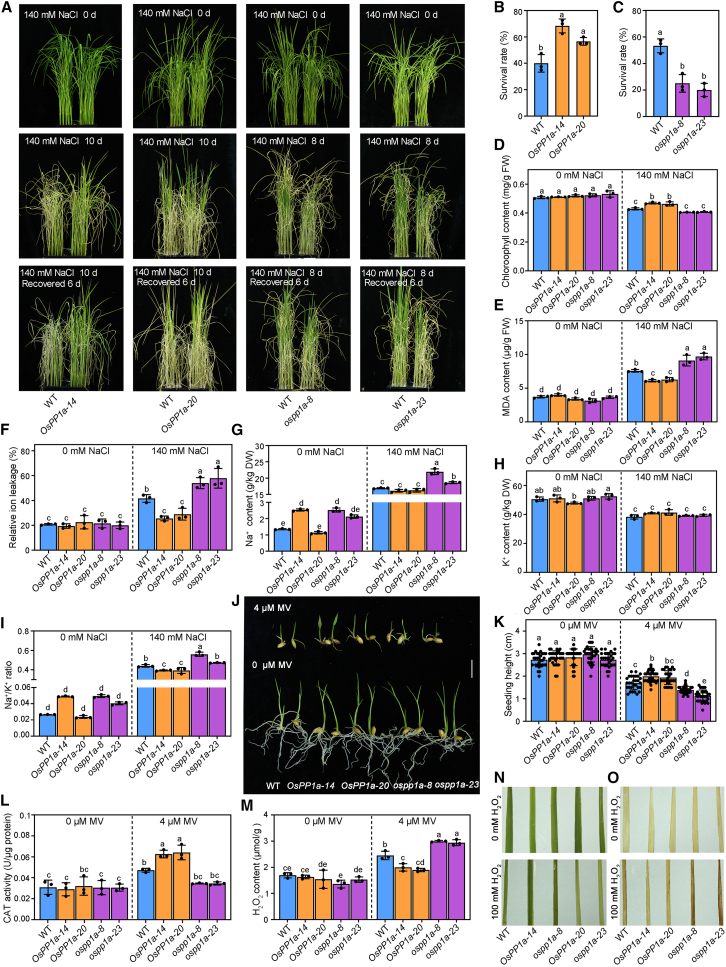
OsPP1a positively regulates salt and oxidative tolerance in rice at the seedling stage. **(A)** Representative images of 15-day-old wild-type (WT), *OsPP1a*-overexpressing (*OsPP1a-14* and *OsPP1a-20*), and *OsPP1a* knockout (*ospp1a-8* and *ospp1a-23*) seedlings grown under salt stress. Seedlings were treated with 140 mM NaCl for 8 or 10 days and then allowed to recover for 6 days. **(B and C)** Seedling survival rates following salt treatment shown in **(A)**. **(D**–**F)** Chlorophyll content **(D)**, MDA content **(E)**, and relative ion leakage **(F)** in leaves of 15-day-old seedlings after treatment with 140 mM NaCl for 24 h. **(G**–**I)** Na^+^**(G)** and K^+^**(H)** contents and Na^+^/K^+^ ratios **(I)** in rice seedlings. **(J**–**M)** Representative images **(J)**, seedling height **(K)**, CAT activity **(L)**, and H_2_O_2_ content **(M)** of rice seedlings subjected to MV stress. Germinated seeds were grown on 1/2 Murashige and Skoog (MS) medium with or without 4 μM MV for 6 days. Scale bar, 1 cm. **(N)** Leaf phenotype of *OsPP1a* transgenic and WT plants at the three-leaf stage under normal conditions or after treatment with 100 mM H_2_O_2_ for 2 days. **(O)** 3,3′-Diaminobenzidine staining of H_2_O_2_ in leaves from untreated and H_2_O_2_-treated *OsPP1a* transgenic and WT plants for 1 day. Data in **(B)–(I)** and **(L)–(M)** are presented as mean ± SD (*n* = 3). Statistically significant differences are indicated by different lowercase letters (*p* < 0.05), as determined by one-way ANOVA for **(B and C)** and two-way ANOVA for **(E)–(I)** and **(K)–(M),** followed by Tukey’s multiple comparisons test.

To further test the effects of OsPP1a in the salt stress response, we treated WT, *OsPP1a*-overexpressing, and knockout seedlings with 140 mM NaCl. Compared with WT, *OsPP1a-*overexpressing lines exhibited significantly higher tolerance to salt stress, with higher survival rates, whereas *ospp1a* mutants were hypersensitive (Figures 1A–1C). Meanwhile, significantly higher chlorophyll content, but lower malondialdehyde (MDA) content and relative ion leakage, were observed in *OsPP1a-*overexpressing lines compared with WT under salt stress, whereas the opposite trends were detected in *ospp1a* mutants (Figures 1D–1F). By contrast, no obvious phenotypic or physiological differences were observed between these transgenic lines and WT under normal conditions (Figures 1A and 1D–1F). Consistent with a previous report (Liao et al., 2016), these results confirm that OsPP1a positively regulates salt tolerance in rice. The effects of OsPP1a on CAT activity and H_2_O_2_ accumulation were further investigated. Under salt stress, overexpression of *OsPP1a* markedly increased CAT activity and reduced H_2_O_2_ accumulation in both shoots and roots compared with WT, whereas knockout of *OsPP1a* produced the opposite results (Supplemental Figure 4). Under normal growth conditions, no significant differences in CAT activity or H_2_O_2_ accumulation were observed between transgenic and WT seedlings. Moreover, *OsPP1a*-overexpressing lines showed a lower Na^+^/K^+^ ratio compared with WT plants under salt stress, whereas *ospp1a* mutants displayed higher Na^+^ accumulation and an increased Na^+^/K^+^ ratio (Figures 1G–1I). These results suggest that OsPP1a enhances salt tolerance by promoting CAT activity to maintain H_2_O_2_ homeostasis, thereby preventing Na^+^ accumulation and mitigating membrane damage under salt stress in rice. To further evaluate the role of OsPP1a in oxidative stress responses, we treated *OsPP1a* transgenic rice seedlings with the oxidative stress inducer methyl viologen (MV). After treatment with 4 μM MV, *OsPP1a*-overexpressing seedlings were taller than WT, whereas *ospp1a* seedlings exhibited a dwarf phenotype (Figures 1J and 1K). Meanwhile, higher CAT activity and lower H_2_O_2_ content were detected in *OsPP1a*-overexpressing seedlings compared with WT, whereas the opposite results were observed in *ospp1a* seedlings (Figures 1L and 1M). By contrast, no noticeable differences in these phenotypic or physiological traits were observed between WT and transgenic lines under normal conditions. Additionally, treatment with 100 mM H_2_O_2_ showed that leaves of *OsPP1a*-overexpressing seedlings exhibited no obvious chlorosis or damage and accumulated less H₂O₂ than WT (Figures 1N and 1O). In contrast, *ospp1a* leaves displayed severe necrosis with higher H_2_O_2_ accumulation. These results indicate that OsPP1a also positively regulates oxidative stress tolerance, with its overexpression enhancing ROS-scavenging capacity in rice.

### OsPP1a physically interacts with CatC *in vitro* and *in vivo*

**Figure 2:**
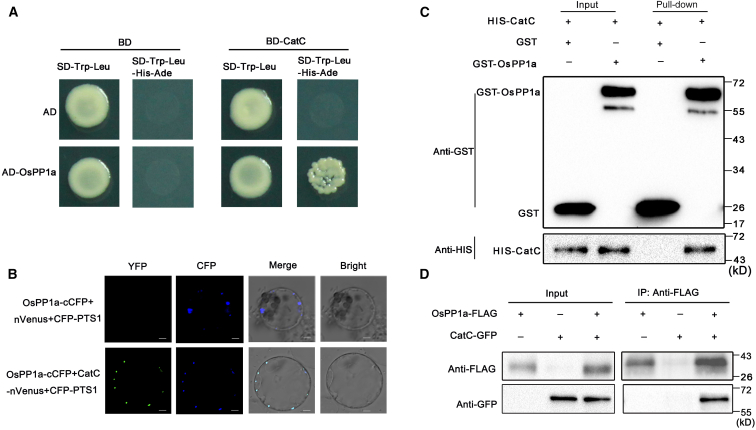
OsPP1a physically interacts with CatC. **(A)** Yeast two-hybrid (Y2H) assay showing the interaction between OsPP1a and CatC. CatC (bait) and OsPP1a (prey) were co-transformed into yeast cells as indicated. Transformants were selected on SD medium lacking leucine (Leu) and tryptophan (Trp), and protein–protein interactions were assessed on SD medium lacking Leu, Trp, histidine (His), and adenine (Ade). Empty vectors pGBKT7 and pGADT7 were used as negative controls. **(B)** Bimolecular fluorescence complementation (BiFC) assay showing the interaction between OsPP1a and CatC in rice protoplasts. OsPP1a-cCFP and CatC-nVenus were co-expressed, with CFP-PTS1 as a peroxisomal marker. Scale bar, 10 μm. **(C)***In vitro* pull-down assay showing the interaction between OsPP1a and CatC. Purified HIS-CatC was incubated with GST-OsPP1a, followed by pull-down using glutathione agarose. Input and eluted fractions were separated by 10% SDS–PAGE and immunoblotted with anti-GST and anti-HIS antibodies. GST was used as a negative control. **(D)** Co-immunoprecipitation (Co-IP) assay showing the interaction between OsPP1a and CatC in *N. benthamiana leaves*. Protein extracts (input) were immunoprecipitated with an anti-FLAG antibody (IP). Immunoblots were probed with anti-FLAG to detect OsPP1a and anti-GFP to detect CatC.

Because OsPP1a was identified as a component of the CatC protein complex, we examined its interaction with CatC using a yeast two-hybrid (Y2H) assay. The full-length OsPP1a and CatC proteins were fused to the GAL4 activation domain in the prey vector (AD–OsPP1a) and the GAL4 DNA-binding domain in the bait vector (BD–CatC), respectively. These constructs were co-transformed into yeast (*Saccharomyces cerevisiae*) to assess potential protein–protein interactions. As expected, OsPP1a physically interacted with CatC in yeast (Figure 2A). Considering the high sequence similarity among CAT family members (CatA, CatB, and CatC) and OsPP1 family members (OsPP1a, OsPP1b, OsPP1c, OsPP1d, and OsPP1e) (Supplemental Figure 1B) (Joo et al., 2014; Liu et al., 2023), we further examined the interactions of OsPP1a with CatA and CatB, as well as those of CatC with OsPP1b, OsPP1c, OsPP1d, OsPP1e, and another protein phosphatase, OsPFA-DSP2 (used as a negative control), using Y2H assays. OsPP1a also interacted with CatA and CatB, showing interaction strengths comparable to that with CatC (Supplemental Figures 5A and 5B). In addition, CatC interacted with OsPP1b, OsPP1c, OsPP1d, and OsPP1e (Supplemental Figures 5C and 5D), but not with the negative control OsPFA-DSP2. Notably, Y2H assays indicated that OsPP1a does not form homodimers (Supplemental Figures 5A and 5B). Given that only CatC and OsPP1a have been implicated in salt stress responses (Joo et al., 2014; Liao et al., 2016; Liu et al., 2023), we focused on characterizing the OsPP1a–CatC interaction. Bimolecular fluorescence complementation (BiFC) assays demonstrated that OsPP1a specifically interacts with CatC in the peroxisomes of rice protoplasts (Figure 2B). Pull-down assays further confirmed that HIS-CatC was efficiently pulled down by glutathione S-transferase (GST)-OsPP1a, but not by GST alone (Figure 2C), indicating a direct interaction *in vitro*. To validate this interaction *in planta*, we conducted co-immunoprecipitation (co-IP) assays by transiently co-expressing OsPP1a-FLAG and CatC-GFP in *Nicotiana benthamiana* leaves. CatC-GFP was co-immunoprecipitated with OsPP1a-FLAG (Figure 2D), demonstrating that OsPP1a interacts with CatC *in vivo*.

PP1 typically functions as a holoenzyme in association with diverse regulatory subunits, many of which contain conserved motifs such as RVxF and SILK that mediate substrate recruitment, PP1 interaction or inhibition, and subcellular targeting. Importantly, some PP1 regulatory subunits also serve as substrates of PP1 (Verbinnen et al., 2017; Choy et al., 2018; Zhang et al., 2020b). To determine whether CatC contains conserved PP1-docking motifs required for OsPP1a binding, we analyzed its sequence and found that CatC lacks the canonical RVxF and SILK motifs but contains an atypical HYVKF motif (amino acids 220–224). Conservation analysis further showed that the HYVKF motif is not conserved among members of the rice CAT family (Supplemental Figure 5F). To assess the role of the HYVKF motif in the OsPP1a–CatC interaction, we generated a series of truncated CatC constructs and examined their interactions with OsPP1a using Y2H assays. The results showed that both N-terminal and C-terminal fragments of CatC, with or without the HYVKF motif, were capable of interacting with OsPP1a (Supplemental Figure 5B), indicating that the OsPP1a–CatC interaction is independent of the HYVKF motif. These findings suggest that CatC may contain an alternative, non-RVxF docking site for OsPP1a.

### OsPP1a dephosphorylates CatC at Thr-292 and enhances its activity

**Figure 3:**
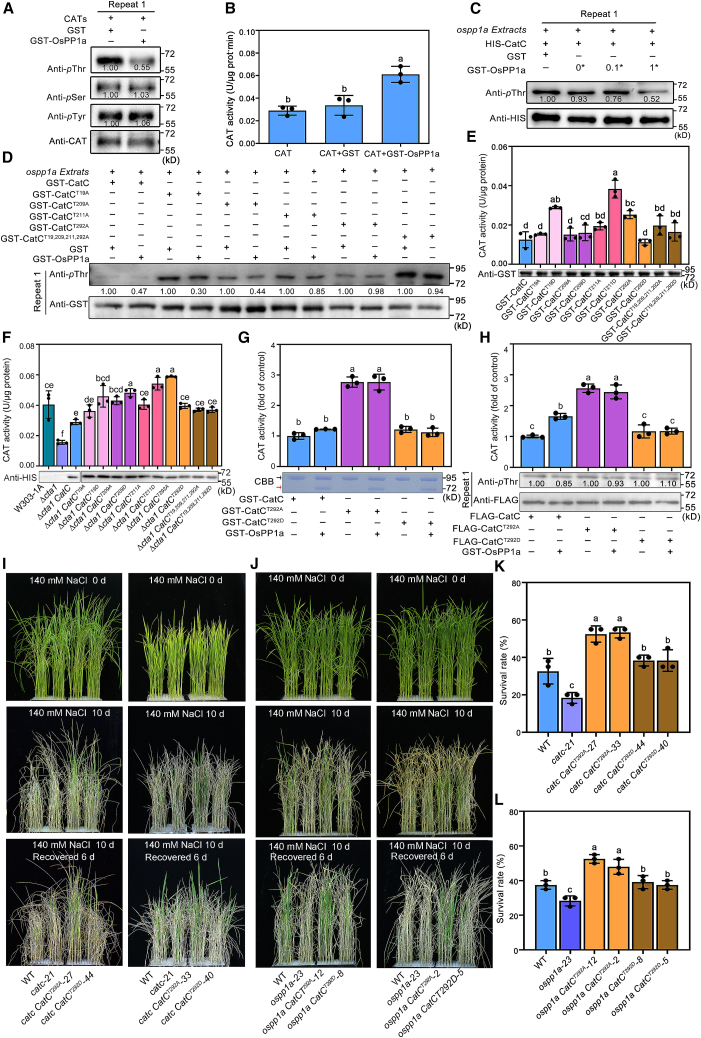
OsPP1a dephosphorylates CatC at Thr-292 and enhances its enzymatic activity *in vitro* and *in vivo*. **(A)** OsPP1a acts as a serine/threonine phosphatase that dephosphorylates phospho-threonine residues on CATs. Phosphorylated CATs were immunoprecipitated using an anti-CAT antibody and incubated with GST-OsPP1a or GST purified from *E. coli*. GST was used as a negative control. Phosphorylation status was determined by immunoblotting using anti-*p*Ser, anti*-p*Thr, and anti-*p*Tyr antibodies. Endogenous CATs were detected with an anti-CAT antibody. Band intensit y ratios (anti-*p*Thr/anti-CAT, anti-*p*Ser/anti-CAT, and anti-*p*Tyr/anti-CAT) for CAT incubated with GST were set to 1. **(B)** OsPP1a enhances CAT activity *in vitro*. CATs were immunoprecipitated from bicinchoninic acid (BCA) assay normalized rice seedling extracts using an anti-CAT antibody and incubated with purified GST-OsPP1a or GST (from *E. coli*) prior to CAT activity measurement. **(C)** OsPP1a dephosphorylates CatC in a dose-dependent manner. *In vitro* dephosphorylation assays showed that increasing amounts of GST-OsPP1a reversed the phosphorylation of HIS-CatC incubated with protein extracts from *ospp1a* mutant seedlings. The amounts of GST-OsPP1a used (0, 1, and 10 μg) are indicated. GST served as a negative control. Band intensity ratios (anti-*p*Thr/anti-HIS) for GST-CatC incubated with GST were set to 1. **(D)***In vitro* dephosphorylation of WT and mutant CatC proteins by GST-OsPP1a. Equal amounts of GST-CatC, GST-CatC^T19A^, GST-CatC^T209A^, GST-CatC^T211A^, GST-CatC^T292A^, and GST-CatC^T19, 209, 211, 292A^ were incubated with extracts from *ospp1a* mutants and treated with GST-OsPP1a or GST. GST was used as a negative control. Band intensity ratios (anti-*p*Thr/anti-HIS) for WT and mutant HIS-CatC proteins incubated with GST were set to 1. **(E)** CAT activities of phospho-mimic and dephospho-mimic CatC proteins expressed and purified from recombinant *E. coli*. Protein loading was verified using an anti-GST antibody. **(F)** CAT activities of *Δcta1* yeast mutant strains expressing phospho-mimic and dephospho-mimic CatC proteins. HIS-tagged CatC variants were expressed in *Δcta1* yeast, and protein loading was confirmed using an anti-HIS antibody. **(G)** Dephosphorylation of CatC at Thr-292 enhances CAT activity *in vitro*. CAT activities of GST-CatC, GST-CatC^T292A^, and GST-CatC^T292D^ purified from *E. coli* were measured in the absence or presence of GST-OsPP1a. CBB, Coomassie Brilliant Blue staining showing protein loading. Black and red arrows indicate GST-CatC and GST-OsPP1a, respectively. **(H)** OsPP1a specifically dephosphorylates CatC at Thr-292 and activates CAT activity. FLAG-CatC, FLAG-CatC^T292A^, and FLAG-CatC^T292D^ were immunoprecipitated from corresponding transgenic lines under normal conditions, and CAT activity was measured with or without GST-OsPP1a. Band intensity ratios (anti-*p*Thr/anti-FLAG) for samples without GST-OsPP1a were set to 1. **(I–L)** Representative images **(I and J)** and survival rates **(K and L)** of seedlings under salt stress. Fifteen-day-old seedlings overexpressing *CatC*^*T292A*^ or *CatC*^*T292D*^ in the *catc-21* (*catc CatC*^*T292A*^ and *catc CatC*^*T292D*^) or *ospp1a-23* (*ospp1a CatC*^*T292*^*A* and *ospp1a CatC*^*T292D*^) backgrounds were treated with 140 mM NaCl for 10 days, followed by a 6-day recovery period. Data in **(E)–(H)**, **(K)**, and **(L)** are presented as mean ± SD (*n* = 3). Statistically significant differences are indicated by different lowercase letters (*p* < 0.05, one-way ANOVA with Tukey’s multiple comparisons test).

To determine whether OsPP1a functions as a phosphatase that dephosphorylates CatC, we immunoprecipitated endogenous CAT proteins from WT rice seedlings using an anti-CAT antibody. The immunoprecipitated complexes were then incubated with GST-OsPP1a purified from *Escherichia coli*. Notably, incubation with GST-OsPP1a led to a significant decrease in phospho-threonine levels, whereas phospho-serine and phospho-tyrosine levels remained relatively unchanged (Figure 3A; Supplemental Figure 6A), indicating that OsPP1a preferentially dephosphorylates phospho-threonine residues of CAT proteins. We next assessed the effect of OsPP1a-mediated dephosphorylation on CAT activity. CAT activity increased by approximately 60% following incubation with GST-OsPP1a compared with untreated controls or samples incubated with GST alone (Figure 3B), suggesting that OsPP1a-mediated dephosphorylation positively regulates CAT enzymatic activity. To further examine the effect of OsPP1a on CatC, we performed *in vitro* kinase/phosphatase assays using purified HIS-CatC as the substrate and protein extracts from *ospp1a* seedlings as the kinase source. HIS-CatC was incubated alone or together with GST-OsPP1a. The addition of GST-OsPP1a reduced the phospho-threonine levels of HIS-CatC in a dose-dependent manner (Figure 3C; Supplemental Figure 6B). These results indicate that OsPP1a specifically dephosphorylates phospho-threonine residues in CatC and enhances its enzymatic activity.

Our previous study identified four phospho-threonine residues in CatC: Thr-19, Thr-209, Thr-211, and Thr-292 (Zhou et al., 2018). To determine which residue is specifically dephosphorylated by OsPP1a, we first synthesized four phosphopeptides corresponding to each of these sites (Supplemental Figure 6C) and incubated them with GST-OsPP1a. Similar to the alkaline phosphatase FastAP used as a positive control, GST-OsPP1a dephosphorylated all four synthetic phosphopeptides (Supplemental Figure 6D). However, synthetic peptides may not fully recapitulate the native conformation of the intact CatC protein and could affect the specificity of OsPP1a. To address this limitation, we performed *in vitro* kinase/phosphatase assays using full-length CatC variants in which threonine (T) residues were substituted with alanine (A) to generate dephospho-mimic mutants (CatC^T19A^, CatC^T209A^, CatC^T211A^, CatC^T292A^, and CatC^T19,209,211,292A^). Notably, only GST-CatC^T292A^ and GST-CatC^T19,209,211,292A^ showed no detectable changes in phospho-threonine levels after incubation with protein extracts from *ospp1a* seedlings and GST-OsPP1a (Figure 3D; Supplemental Figure 6E), indicating that OsPP1a specifically dephosphorylates CatC at Thr-292 *in vitro*. To assess the functional impact of phosphorylation on CAT activity, we generated phospho-mimic variants by substituting threonine residues with aspartic acid (D) (CatC^T19D^, CatC^T209D^, CatC^T211D^, CatC^T292D^, and CatC^T19,209,211,292D^). Both phospho-mimic and dephospho-mimic variants of CatC, fused to GST at the N terminus, were expressed and purified from *E. coli* for *in vitro* CAT activity assays. Among these, only the dephospho-mimic variant GST-CatC^T292A^ exhibited markedly higher activity than its phospho-mimic counterpart GST-CatC^T292D^ and the WT protein GST-CatC (Figure 3E). This result is consistent with previous findings showing that OsPP1a promotes CAT activity through dephosphorylation of threonine residues (Figures 3A–3C). To further validate these observations *in vivo, CatC variants were expressed in the CAT-deficien*t yeast mutant *Δcta1*, and CAT activity and H_2_O_2_ levels were measured (Supplemental Figures 7A and 7B). Consistently, the *Δcta1* CatC^T292A^ strain exhibited significantly higher CAT activity and lower H_2_O_2_ levels than the *Δcta1* CatC^T292D^ strain (Figure 3F; Supplemental Figure 7C), further confirming that dephosphorylation of Thr-292 enhances CAT activity. Moreover, *in vitro* assays showed that the dephospho-mimic variant CatC^T292A^ displayed the highest CAT activity regardless of the presence of GST-OsPP1a (Figure 3G). Together, these results demonstrate that OsPP1a specifically dephosphorylates CatC at Thr-292, thereby enhancing CAT activity. Notably, Thr-292 is highly conserved within the CAT family (Supplemental Figure 8D), suggesting that it represents a key regulatory site for reversible phosphorylation controlling CAT function in plants.

To investigate the biological significance of Thr-292 *in planta*, we overexpressed *CatC* variants (*CatC*, *CatC*^*T292A*^, and *CatC*^*T292D*^) with N-terminal FLAG tags in the *ospp1a-23* mutant background. These variants were immunoprecipitated from transgenic lines treated with 140 mM NaCl for 30 min and subsequently incubated with GST-OsPP1a. FLAG-CatC showed a decreased phospho-threonine level and increased CAT activity after incubation with GST-OsPP1a, whereas FLAG-CatC^T292A^ and FLAG-CatC^T292D^ maintained relatively stable phospho-threonine levels and CAT activities (Figure 3H; Supplemental Figure 6F). Notably, FLAG-CatC^T292A^ exhibited the highest CAT activity regardless of GST-OsPP1a treatment. These results further confirm that OsPP1a dephosphorylates CatC at Thr-292 and positively regulates CAT activity. In addition, we generated *CatC* knockout mutants (Supplemental Figure 8A) and individually overexpressed *CatC*, *CatC*^*T292A*^, and *CatC*^*T292D*^ in the *catc-21* or *ospp1a-23* backgrounds (Supplemental Figures 8B–8E). *In vivo* CAT activity and H_2_O_2_ content were then measured in these lines. Compared with their respective controls (*catc-21* and *ospp1a-23*), lines overexpressing *CatC*^*T292A*^ (*catc CatC*^*T292A*^ and *ospp1a CatC*^*T292A*^) exhibited the highest CAT activity and lowest H₂O₂ levels, whereas lines overexpressing *CatC*^*T292D*^ (*catc CatC*^*T292D*^ and *ospp1a CatC*^*T292D*^) showed only moderate increases in CAT activity and slight reductions in H_2_O_2_ accumulation (Supplemental Figures 9A–9D). These results indicate that, in the absence of endogenous CatC and OsPP1a, dephosphorylation of CatC at Thr-292 enhances CAT activity and promotes efficient H₂O₂ scavenging in rice. To assess the effect of the phosphorylation state of CatC at Thr-292 on salt stress responses, we treated these transgenic seedlings with 140 mM NaCl. Both *catc CatC*^*T292A*^ and *ospp1a CatC*^*T292A*^ lines exhibited the strongest salt tolerance with the highest survival rates (Figures 3I–3L), indicating that dephosphorylation of CatC at Thr-292 enhances salt tolerance in rice. Furthermore, CatC^T19,209,211,292A^ and CatC^T19,209,211,292D^ were individually overexpressed in the *catc-21* and *ospp1a-23* backgrounds (Supplemental Figures 8B–8E), and their responses to salt stress were analyzed. Both variants increased the sensitivity of rice seedlings to salt stress (Supplemental Figures 9E–9H), further supporting that OsPP1a specifically targets Thr-292, rather than Thr-19, Thr-209, or Thr-211. Considering their CAT activity and H_2_O_2_ profiles (Supplemental Figures 9A–9D), we propose that the enhanced salt tolerance observed in *CatC*^*T292A*^ transgenic lines results from increased CAT activity and more efficient H₂O₂ scavenging.

### OsPP1a-mediated dephosphorylation stabilizes CatC by inhibiting its ubiquitination and degradation

**Figure 4:**
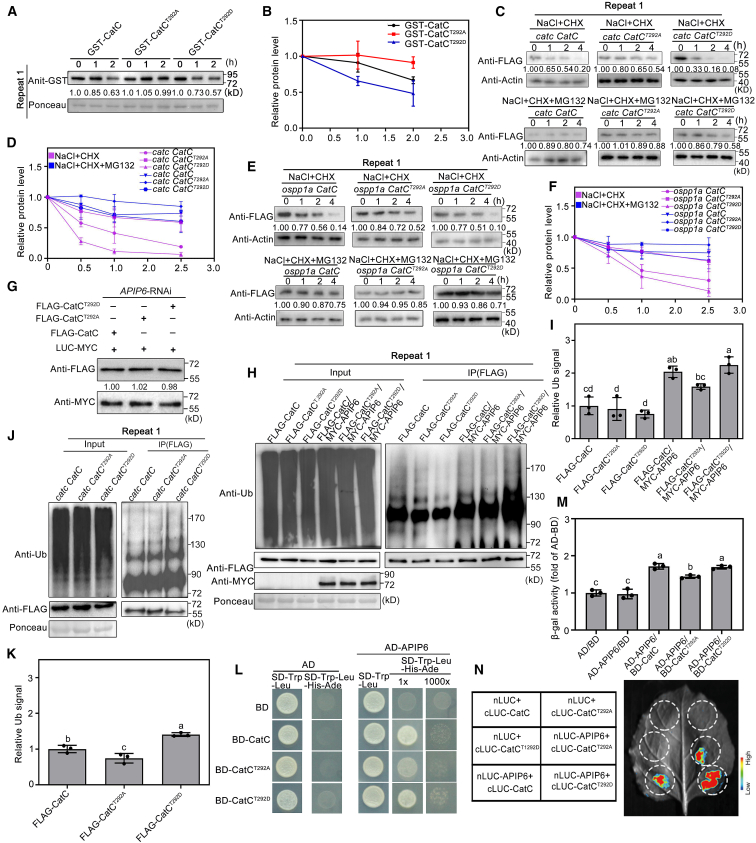
OsPP1a-mediated dephosphorylation stabilizes CatC by inhibiting its ubiquitination and degradation. **(A and B)** Protein stability of CatC variants **(A)** and corresponding quantification **(B)** in a cell-free degradation system. Protein loading was verified by Ponceau S staining. Band intensity ratios (anti-GST/Ponceau) for GST-CatC variants prior to incubation with the cell-free extract from WT rice seedlings were set to 1. Each assay was biologically replicated three times. Data are presented as mean ± SD (*n* = 3). **(C and E)** Representative immunoblots showing protein levels of FLAG-CatC variants in *catc* mutant **(C)** or *ospp1a* mutant **(E)** backgrounds under salt stress. Five-day-old seedlings overexpressing *FLAG-CatC*, *FLAG-CatC*^*T292A*^, or *FLAG-CatC*^*T292D*^ in the *catc-21* (*catc CatC*, *catc CatC*^*T292A*^, *catc CatC*^*T292D*^) or *ospp1a-23* (*ospp1a CatC*, *ospp1a CatC*^*T292A*^, *ospp1a CatC*^*T292D*^) backgrounds were treated with 50 μM cycloheximide (CHX) and/or 50 μM MG132 for the indicated times. Total proteins were extracted, and the abundance of CatC variants was detected using an anti-FLAG antibody. Actin served as an internal control. Each assay was biologically replicated three times. **(D and F)** Relative protein levels of FLAG-CatC variants corresponding to **(C) and (E)**. Band intensity ratios (anti-FLAG/anti-actin) at 0 h (before salt treatment) were set to 1. Data are presented as mean ± SD (*n* = 3). **(G)** Representative immunoblots showing protein levels of CatC variants in *APIP6*-RNAi protoplasts. *FLAG-CatC*, *FLAG-CatC*^*T292A*^, and *FLAG-CatC*^*T292D*^ were transiently expressed in protoplasts derived from *APIP6*-RNAi plants with LUC-MYC as an internal control. Band intensity ratios (anti-FLAG/anti-MYC) for FLAG-CatC were set to 1. Each assay was biologically replicated three times. **(H and I)** Ubiquitination assays showing that dephosphorylation of CatC at Thr-292 inhibits its APIP6-mediated ubiquitination in *N. benthamiana*. *FLAG-CatC*, *FLAG-CatC*^*T292A*^, and *FLAG-CatC*^*T292D*^ were expressed alone or co-expressed with *MYC-APIP6* in *N. benthamiana* leaves for 48 h, followed by treatment with 50 μM MG132 for 6 h. Quantification of ubiquitinated CatC variants in **(H)** is shown in **(I)**. **(J and K)** Ubiquitination of CatC variants in *catc* mutant seedlings under salt stress. Fifteen-day-old catc seedlings overexpressing *FLAG-CatC*, *FLAG-CatC*^*T292A*^, and *FLAG-CatC*^*T292D*^ were treated with 50 μM MG132 followed by 140 mM NaCl for 4 h. Quantification of ubiquitinated CatC variants in **(J)** is shown in **(K)**. Ubiquitinated CatC variants in **(H) and (J)** were detected using an anti-ubiquitin (anti-Ub) antibody, and Ponceau S staining was used as a loading control. Ubiquitination signals were quantified using ImageJ and normalized to the corresponding CatC protein levels in the IP. Each assay was biologically replicated three times. **(L and M)** Dephosphorylation of CatC at Thr-292 weakens its interaction with APIP6, as shown by Y2H assays **(L)** and β-galactosidase activity **(M)**. β-galactosidase activity is presented as fold change relative to the AD-BD control. **(N)** Luciferase complementation imaging (LCI) assays showing interactions between APIP6 and CatC or its variants (CatC^T292A^ and CatC^T292D^) in *N. benthamiana* leaves. Data in **(I), (K), and (M)** are presented as mean ± SD (*n* = 3). Statistically significant differences are indicated by different lowercase letters (*p* < 0.05, one-way ANOVA with Tukey’s multiple comparisons test).

Enzymatic activity is often influenced by protein stability, and proteins with greater stability typically exhibit higher activity (Yang and Guo, 2018). To determine the effect of CatC Thr-292 phosphorylation on its stability, we performed a cell-free degradation assay. Recombinant GST-CatC, GST-CatC^T292A^, and GST-CatC^T292D^ proteins purified from *E. coli* were incubated with total protein extracts from WT rice plants and analyzed by immunoblotting using an anti-GST antibody. As incubation time increased, GST-CatC and GST-CatC^T292D^ showed markedly increased degradation, whereas GST-CatC^T292A^ exhibited no significant degradation (Figures 4A and 4B; Supplemental Figure 10A), suggesting that dephosphorylation at Thr-292 stabilizes CatC. To further investigate the effect of OsPP1a-mediated dephosphorylation on CatC stability under salt stress in rice, we treated *catc* and *ospp1a* mutant lines overexpressing *FLAG-CatC* variants with the protein synthesis inhibitor cycloheximide (CHX), the proteasome inhibitor MG132, and 140 mM NaCl. The degradation of FLAG-CatC and FLAG-CatC^T292D^ increased markedly with prolonged salt and CHX treatment, whereas FLAG-CatC^T292A^ showed only a slight increase in degradation (Figures 4C–4F; Supplemental Figures 10B and 10C), consistent with the *in vitro* results. Notably, in the *catc mutant background,* the degradation rate of FLAG-CatC^T292D^ was significantly faster than that of FLAG-CatC, whereas this difference was not significant in the *ospp1a* mutant background (Figures 4D and 4F). Furthermore, when treated with salt, CHX, and MG132, the degradation of FLAG-CatC, FLAG-CatC^T292A^, and FLAG-CatC^T292D^ was markedly reduced in both *catc* and *ospp1a* backgrounds compared with treatment with salt and CHX alone, indicating that CatC degradation is inhibited by MG132 and is mediated by the ubiquitin–26S proteasome pathway.

CAT has been reported to be ubiquitinated by the E3 ubiquitin ligase APIP6 and degraded via the 26S proteasome pathway (You et al., 2022). Therefore, we further investigated the effect of OsPP1a-mediated dephosphorylation of CatC on its APIP6-mediated degradation in rice. We transiently expressed *FLAG-CatC* variants and analyzed their protein levels in protoplasts derived from *APIP6*-RNAi plants. No significant differences in protein abundance were observed among FLAG-CatC, FLAG-CatC^T292A^, and FLAG-CatC^T292D^ in *APIP6*-RNAi protoplasts (Figure 4G). We next co-expressed *HIS-CatC* variants with *MYC-APIP6* in rice plants (Supplemental Figures 8F and 8G) and examined their protein stability, CAT activity, and H_2_O_2_ content. HIS-CatC^T292A^ was more stable than HIS-CatC^T292D^ and HIS-CatC when co-expressed with MYC-APIP6 (Supplemental Figures 11A and 11B). Consistently, *APIP6 CatC*^*T292A*^ lines exhibited the highest CAT activity and lowest H₂O₂ levels, whereas *APIP6 CatC*^*T292D*^ lines showed no obvious differences compared with *APIP6 CatC* lines (Supplemental Figures 11C and 11D). These results indicate that dephosphorylation of CatC at Thr-292 inhibits APIP6-mediated degradation, thereby enhancing CAT stability and activity, as well as H_2_O_2_-scavenging capacity, in rice.

To investigate the effect of CatC Thr-292 phosphorylation on its ubiquitination status, we transiently expressed *FLAG-CatC* variants either alone or together with *MYC-APIP6* in *N. benthamiana* leaves and assessed ubiquitination levels by immunoblotting using an anti-ubiquitin antibody. At comparable CatC protein levels, FLAG-CatC^T292A^ exhibited significantly lower ubiquitination, whereas FLAG-CatC^T292D^ showed slightly higher ubiquitination than FLAG-CatC when co-expressed with APIP6-MYC (Figures 4H and 4I; Supplemental Figure 12A). Consistently, in the *catc mutant background under salt stress,* the ubiquitination level of FLAG-CatC^T292A^ was markedly lower than those of FLAG-CatC^T292D^ and FLAG-CatC (Figures 4J and 4K; Supplemental Figure 12B). These results indicate that dephosphorylation of CatC at Thr-292 suppresses its ubiquitination. We next examined whether the phosphorylation status of Thr-292 affects the interaction between CatC and APIP6. Y2H assays showed that CatC^T292A^ exhibited a markedly weaker interaction with APIP6 than CatC and CatC^T292D^ (Figures 4L and 4M). Similar results were obtained from luciferase complementation imaging (LCI) assays in *N. benthamiana* leaves (Figure 4N). These findings indicate that dephosphorylation of Thr-292 weakens the interaction between CatC and APIP6, thereby inhibiting APIP6-mediated ubiquitination and degradation of CatC. Although subcellular localization and polymerization of CAT can also influence its enzymatic activity (Al-Hajaya et al., 2022; Wang et al., 2023b; Liu et al., 2023), our results show that phosphorylation at Thr-292 does not affect CatC peroxisomal localization or polymerization (Supplemental Figure 13).Collectively, these findings demonstrate that OsPP1a-mediated dephosphorylation of CatC at Thr-292 impairs its interaction with APIP6, thereby stabilizing CatC by inhibiting APIP6-mediated ubiquitination and degradation in rice.

### OsPP1a plays a vital role in the response to salt stress in rice

**Figure 5:**
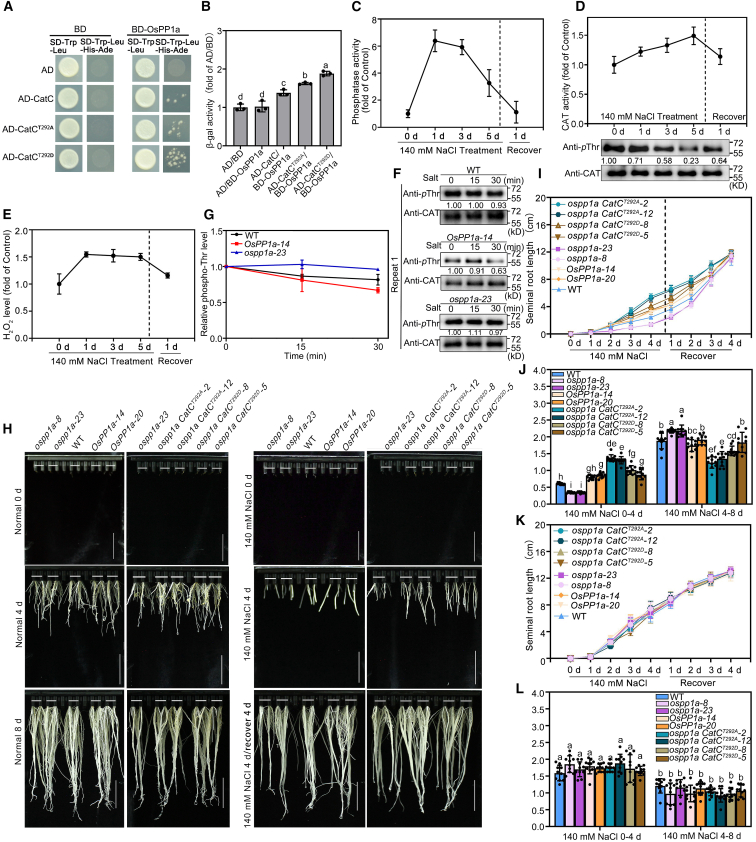
OsPP1a plays a vital role in the response to salt stress in rice. **(A and B)** Phosphorylation at Thr-292 of CatC enhances its interaction with OsPP1a, as shown by Y2H **(A)** and *β*-galactosidase **(B)** assays in yeast. **(C)** OsPP1a phosphatase activity is activated by salt stress but inhibited after stress recovery. The FLAG-OsPP1a fusion protein was immunoprecipitated from *OsPP1a-14* plants treated with 140 mM NaCl for the indicated times and used for phosphatase activity assays. **(D)** Salt stress decreases the phospho-threonine level of CATs while enhancing their enzymatic activity, and the opposite trend is observed after recovery. Endogenous CATs were immunoprecipitated from *OsPP1a-14* plants treated with 140 mM NaCl for the indicated times using an anti-CAT antibody. Phosphorylation levels were detected using an anti-*pThr antibody. Relative CAT* activity and anti-*p*Thr/anti-CAT ratios at 0 days (no NaCl treatment) were set to 1. **(E)** H_2_O_2_ content is slightly increased under salt stress but decreases after recovery. H_2_O_2_ levels were measured in *OsPP1a-14* plants treated with 140 mM NaCl for the indicated times. The relative H_2_O_2_ content at 0 days was set to 1. **(F and G)** Phospho-threonine levels of CATs **(F)** and their quantification **(G)** in WT and *OsPP1a* transgenic rice plants. Endogenous CATs were immunoprecipitated from WT, *OsPP1a-14*, and *ospp1a-23* seedlings after 140 mM NaCl treatment for the indicated times and analyzed by immunoblotting. Band intensity ratios (anti-*p*Thr/anti-CAT) at 0 min were set to 1. **(H)** Seminal root growth analysis of the indicated rice seedlings during the transition from salt stress to normal conditions. Seeds were germinated in 140 mM NaCl for 4 days and then transferred to water for an additional 4 days. Seedlings grown in water only served as controls. Bar, 4 cm.**(I**–**L)** Seminal root length **(I and K)** and average growth rate **(J and L)** of the seedlings shown in **(H)**. Thirty seedlings per line were analyzed. Data in **(B)–(E) and (G)** are presented as mean ± SD (*n* = 3). Statistically significant differences are indicated by different lowercase letters (*p* < 0.05, two-way ANOVA for **(J) and (L)** with Tukey’s multiple comparisons test).

Although activation of stress responses can significantly improve survival under salt stress, constitutive tolerance is often costly and compromises growth and yield in crops (Zhang et al., 2020a; Zhao et al., 2021; Liu et al., 2023). Therefore, salt stress responses must be tightly regulated to enable rapid adaptation while minimizing growth penalties once stress is alleviated. We first examined the effect of Thr-292 phosphorylation on the OsPP1a–CatC interaction using Y2H assays. The phospho-mimic form CatC^T292D^ exhibited the strongest interaction with OsPP1a compared with CatC and CatC^T292A^ (Figures 5A and 5B), suggesting that phosphorylation at Thr-292 promotes the interaction between OsPP1a and CatC and may play an important role in salt stress responses. To further assess the effect of salt stress on OsPP1a phosphatase activity *in planta*, we measured the activity of OsPP1a immunoprecipitated from *OsPP1a*-overexpressing lines treated with 140 mM NaCl for 0, 1, 3, and 5 days, followed by a 1-day recovery period. OsPP1a exhibited a clear and transient activation in response to salt stress, with a sharp increase on day 1 and a return to baseline after recovery (Figure 5C). Consistently, CAT activity increased, phospho-threonine levels decreased, and H₂O₂ levels showed a slight increase under salt stress, followed by reduced CAT activity and H_2_O_2_ levels after recovery (Figures 5D and 5E). Based on these observations, we propose that salt stress activates OsPP1a, which dephosphorylates CatC at Thr-292, thereby stabilizing CatC and enhancing CAT activity to scavenge excess H_2_O_2_ and improve salt tolerance in rice. Because early responses are critical for plant adaptation to saline conditions (Liu et al., 2023), we further examined CAT phospho-threonine levels under short-term salt stress. As expected, phospho-threonine levels of CATs were significantly decreased in *OsPP1a*-overexpressing seedlings during the early stages of salt stress, whereas no obvious changes were observed in WT or *ospp1a* seedlings (Figures 5F and 5G; Supplemental Figure 12C). These results indicate that OsPP1a also functions in the early response to salt stress. Together, these findings demonstrate that activation of OsPP1a and its mediated dephosphorylation of CATs are essential for active salt stress responses in rice.

Rice seminal roots are hypersensitive to salt stress, making them an excellent model for studying salt-stress responses (Zou et al., 2021; Liu et al., 2023). To investigate the role of OsPP1a in balancing salt tolerance and growth, we analyzed seminal root growth during the transition from salt stress to normal growth conditions. Under salt stress, *OsPP1a*-overexpressing lines exhibited higher seminal root growth rates than WT seedlings, whereas those of *ospp1a* mutants were lower (Figures 5H–5J), indicating that OsPP1a positively regulates root growth and salt tolerance. Meanwhile, *ospp1a CatC*^*T292A*^ lines showed higher seminal root growth rates than *ospp1a CatC*^*T292D*^ and *ospp1a-23* seedlings under salt stress, suggesting that dephosphorylation of CatC at Thr-292 promotes root growth and salt tolerance. Notably, *ospp1a CatC*^*T292A*^ lines displayed the highest seminal root growth rates among all genotypes tested under salt stress (Figures 5H–5J), implying that even when upstream stress signal transduction via dephosphorylation is disrupted in *ospp1a* mutants, the dephosphorylation-mimic form CatC^T292A^ can still promote root growth and salt tolerance. It should be noted that, because CatC^T292D^ retained partial CAT activity and was overexpressed (Figures 3E–3H; Supplemental Figures 9A–9D), *ospp1a CatC*^*T292D*^ lines also showed higher seminal root growth rates than WT seedlings, although lower than those of *ospp1a CatC*^*T292A*^ lines under salt stress. Upon removal of salt stress, *ospp1a* mutants exhibited the highest, whereas *ospp1a CatC*^*T292A*^ lines showed the lowest seminal root growth rates among all genotypes tested (Figures 5H–5J), suggesting that inhibition of OsPP1a activity and phosphorylation of CatC at Thr-292 are required for the transition from salt stress to normal growth. By contrast, no noticeable differences in seminal root growth rates were observed among these transgenic lines and WT under normal growth conditions (Figures 5H–5L). Together, these results demonstrate that OsPP1a plays a crucial role in the salt-stress response and maintains a balance between salt tolerance and growth in rice.

### OsPP1a improves rice grain yield under salt stress

**Figure 6:**
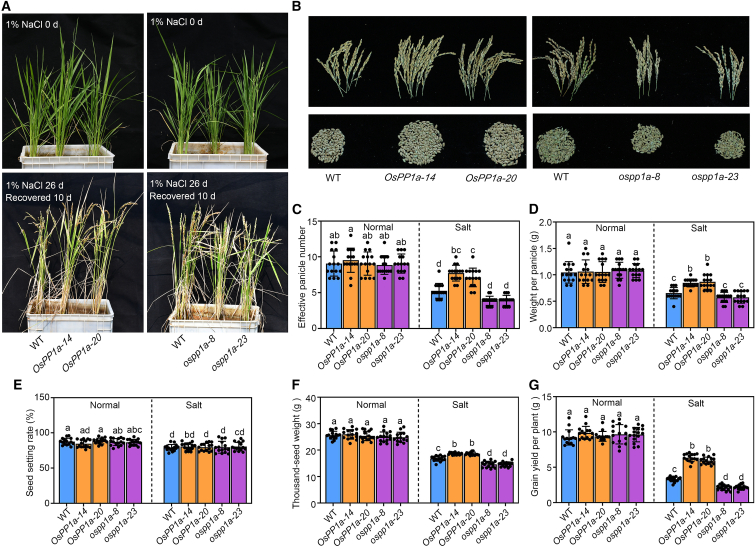
OsPP1a improves rice grain yield under salt stress at the reproductive stage. **(A)** Phenotypic comparison of *OsPP1a-*overexpressing lines, *ospp1a* mutants, and WT plants under salt stress. Salt stress was applied at the panicle development stage by irrigating plants with 1% (approximately 170 mM) NaCl for 26 days, followed by a 10-day recovery period under normal irrigation before harvest. **(B)** Representative panicles (top) and grains (bottom) from a single plant of each genotype shown in **(A)**, collected after the 10-day recovery period. **(C**–**G)** Agronomic trait analysis under salt-stress and normal conditions, including effective panicle number per plant **(C)**, weight per panicle **(D)**, seed-setting rate **(E)**, thousand-seed weight **(F)**, and grain yield per plant **(G)** in *OsPP1a-*overexpressing, *ospp1a*, and WT plants. Measurements were taken after the 26-day salt treatment and 10-day recovery period or at maturity under normal conditions. Data in **(C)–(G)** are presented as mean ± SD. Statistically significant differences are indicated by different lowercase letters (*p* < 0.05, two-way ANOVA with Tukey’s multiple comparisons test).

As one of the most important cereal crops, rice breeding aims to achieve high grain yield under diverse conditions. Although OsPP1a has been shown to significantly enhance growth under salt stress and positively regulate salt tolerance at the seedling stage (Figure 1), it remains important to determine whether OsPP1a also improves agronomic traits and salt tolerance during the reproductive stage. To address this, we evaluated the effects of OsPP1a on agronomic traits, particularly grain yield, during the reproductive stage under salt stress. *OsPP1a*-overexpressing lines, *ospp1a* mutants, and WT plants were grown in plastic pots under normal conditions and then exposed to 1% (w/v; approximately 170 mM) NaCl at the panicle development stage. No noticeable differences in growth vigor or agronomic traits were observed between transgenic and WT plants before salt treatment (Figure 6; Supplemental Figure 14). After 26 days of salt treatment followed by 10 days of recovery, *OsPP1a*-overexpressing lines retained more green leaves than WT plants, whereas *ospp1a* mutants had fewer green leaves (Figure 6A), indicating that OsPP1a also enhances growth under salt stress at the reproductive stage. Moreover, compared with WT plants, *OsPP1a*-overexpressing lines showed marked increases in effective panicle number, weight per panicle, and thousand-seed weight, whereas *ospp1a* lines exhibited sharp decreases in these traits after salt treatment (Figures 6B–6G). Notably, grain yield per plant in *OsPP1a*-overexpressing lines increased by approximately 65% compared with WT plants under salt stress, while that of *ospp1a* lines was markedly lower (Figure 6G). In contrast, no significant differences in these traits were observed between WT and transgenic plants under normal conditions (Figures 6C–6G; Supplemental Figure 14). Collectively, these results demonstrate that OsPP1a enhances salt tolerance during the reproductive stage and mitigates yield loss in rice under salt stress.

## Discussion

### OsPP1a positively regulates salt and oxidative stress tolerance in rice

The increasing salinity of agricultural soils, driven by irrigation practices, climate change, and natural processes (Munns et al., 2020; Liang et al., 2024), poses a significant challenge to crop cultivation. Rice is a glycophyte with limited tolerance to high salinity, necessitating the enhancement of salt tolerance across developmental stages to ensure yield stability under saline conditions. Protein phosphatases play critical roles in regulating signaling networks through the dephosphorylation of proteins targeted by kinases. Among these, PP1 is a predominant member of the Ser/Thr phosphoprotein phosphatase family and is integral to plant growth, development, and stress responses (Shi, 2009). For example, StPP1c interacts with the *Phytophthora infestans* effector Pi04314 to promote late blight disease in potato (*Solanum tuberosum*) (Boevink et al., 2016). AtTOPP4 enhances growth and development by attenuating phytochrome-dependent light responses via dephosphorylation of PIF5 in Arabidopsis (Yue et al., 2016), whereas AtTOPP8 and TOPP9 regulate cell-wall integrity during tip growth (Christina et al., 2018). In addition, AtTOPP4 activity is inhibited by the regulatory subunit PP1R3, which also affects its nuclear localization and abscisic acid (ABA) response (Zhang et al., 2020b). In this study, we identified the PP1 phosphatase OsPP1a from the CatC protein complex in rice using immunoprecipitation–mass spectrometry assays (Supplemental Table 1). Notably, among CAT family members, CatC uniquely and positively regulates salt tolerance in rice (Zhou et al., 2018; Deng et al., 2021). We found that OsPP1a transcripts are predominantly expressed in leaves and are induced by multiple abiotic stresses, including salt, alkali, osmotic, and oxidative stresses (Supplemental Figure 2). These findings are consistent with previous reports showing that *OsPP1a* overexpression enhances salt tolerance in rice by promoting ROS scavenging, although its specific substrates and underlying mechanisms remain unclear (Liao et al., 2016). Here, we demonstrate that *OsPP1a*-overexpressing lines exhibit enhanced tolerance to both salt and oxidative stresses, whereas *ospp1a* mutants are hypersensitive (Figure 1). Interestingly, salt-response analyses of *ospp1a* and *ospp1a ospp1b* mutants reveal functional redundancy between OsPP1a and its homolog OsPP1b, indicating a synergistic role in enhancing salt tolerance (Supplemental Figures 3E and 3G). This is further supported by the characterization of *ospp1a ospp1c* double mutants, which display severe dwarfism and sterility (Supplemental Figure 3E). However, *ospp1a(−/−) ospp1c(+/−)* heterozygotes exhibit no obvious difference in salt response compared with *ospp1a* mutants (Supplemental Figures 3F and 3G), suggesting that OsPP1c primarily regulates growth and development in rice. Collectively, these findings establish OsPP1a as a positive regulator of salt and oxidative stress tolerance and suggest that increasing *OsPP1a* expression may represent a promising strategy for breeding salt-tolerant rice varieties.

ROSs are often regarded as toxic byproducts of stress; however, they also function as crucial signaling molecules that mediate stress responses by integrating transcriptional, hormonal, and metabolic reprogramming (Mittler et al., 2004, 2022; Schmidt et al., 2013). Therefore, maintaining ROS homeostasis, rather than simply minimizing ROS levels, is critical for improving abiotic stress tolerance. Consistent with this concept, several rice regulators enhance salt tolerance by limiting excessive ROS accumulation, including the calcium-dependent protein kinases OsCPK4 and OsCPK12, the receptor-like kinase OsSIK1 (Ouyang et al., 2010; Asano et al., 2012; Campo et al., 2014), and the transcription factor OsZFP213 (Zhang et al., 2018). In addition, heterologous expression of the fungal glutamate dehydrogenase gene *AcGDH* alleviates ROS accumulation and improves drought and alkali tolerance in rice (Yan et al., 2021). CAT is a central antioxidant enzyme that protects plant cells from stress by maintaining H_2_O_2_ homeostasis, and its activity is tightly regulated through reversible protein phosphorylation (Zou et al., 2015; Zhou et al., 2018; Liu et al., 2023; Bai et al., 2024). In this study, we found that OsPP1a and CatC localize to the peroxisome, where they physically interact (Figure 2; Supplemental Figures 2A and 13A). This localization contrasts with previous reports for OsPP1a and its Arabidopsis homolog AtTOPP4, which were shown to reside in the cytoplasm and nucleus (Zhang et al., 2020b; Qin et al., 2024). This difference suggests that the subcellular distribution of PP1 phosphatases may be influenced more by their interacting partners and environmental conditions than by fixed targeting signals. Stress-induced peroxisomal targeting of signaling proteins represents a common mechanism to ensure spatial specificity in ROS regulation (Mittler, 2017; Al-Hajaya et al., 2022). Consistently, we found that OsPP1a is predominantly recruited to the peroxisome under salt stress, highlighting the importance of spatial regulation in ROS homeostasis.

Most PP1s do not exist in cells as free subunits; instead, they typically recruit substrates by recognizing conserved motifs such as RVxF or SILK (Verbinnen et al., 2017; Choy et al., 2018). However, CatC contains only an atypical HYVKF motif, which is not conserved within the CAT family and does not mediate the OsPP1a–CatC interaction (Supplemental Figures 5F and 5G). This finding suggests that CatC harbors an alternative, non-RVxF docking site for OsPP1a. The mechanisms underlying the interaction between OsPP1a and CatC, as well as their peroxisomal targeting, warrant further investigation. Additionally, we found that OsPP1a interacts not only with CatC but also with CatA and CatB (Supplemental Figure 5A), indicating a broader regulatory network. Functional assays further demonstrated that OsPP1a specifically dephosphorylates CatC at Thr-292, thereby enhancing its activity (Figures 3A–3H). Importantly, overexpression of *CatC*^*T292A*^ in both *catc* and *ospp1a* mutants (*catc CatC*^*T292A*^ and *ospp1a CatC*^*T292A*^) significantly improved salt tolerance, accompanied by the highest CAT activity and the lowest H_2_O_2_ accumulation (Figures 3I–3L; Supplemental Figures 9A–9D). These results demonstrate that OsPP1a activates CatC through dephosphorylation, thereby enhancing its H_2_O_2_-scavenging capacity, maintaining H_2_O_2_ homeostasis, and promoting salt and oxidative stress tolerance in rice. Furthermore, *OsPP1a-*overexpressing lines exhibited lower Na^+^ accumulation and maintained a reduced Na^+^/K^+^ ratio (Figures 1G–1I), both of which are key determinants of salt tolerance, although the underlying mechanisms remain to be elucidated. Importantly, *OsPP1a*-overexpressing lines showed significant increases in effective panicle number, weight per panicle, thousand-seed weight, and grain yield per plant under salt stress during the reproductive stage (Figure 6), with no noticeable differences observed under normal conditions (Figure 6; Supplemental Figure 14). Together, these findings suggest that *OsPP1a* is a promising candidate gene for improving rice yield under salt stress without compromising growth.

### OsPP1a stabilizes CatC by inhibiting APIP6-mediated ubiquitination and degradation

ROS play a dual role in plant growth and stress responses. At low steady-state levels, they act as signaling molecules that promote growth and adaptive stress responses. Conversely, excessive ROS accumulation is toxic, impairing cellular functions and leading to oxidative stress (Suzuki et al., 2012; Mittler, 2017). Thus, maintaining ROS homeostasis is essential for normal cellular function. CAT is a key H_2_O_2_-scavenging enzyme and a phosphoprotein whose activity is modulated by reversible phosphorylation mediated by kinases and phosphatases. To date, the mechanisms regulating CAT activation by kinases and deactivation by phosphatases have been partially elucidated. CAT activity is positively regulated by kinases such as PKCδ (Mhamdi et al., 2012; Rafikov et al., 2014), CPK8/12 (Zou et al., 2015; Wang et al., 2023a), and BAK1 (Zhang et al., 2020c), whereas it is negatively regulated by phosphatases PC1 (Liu et al., 2023) and PP2C1 (Bai et al., 2024). Notably, we previously showed that PC1 dephosphorylates CatC at Ser-9, thereby inhibiting its tetramerization and negatively regulating salt tolerance in rice (Liu et al., 2023). PP2C1 has also been reported to dephosphorylate CAT at Ser-112, reducing its activity and stress tolerance in cassava (Bai et al., 2024). Although we previously reported that dephosphorylation of CatC at Ser-18 promotes its tetramerization and activity in rice, the relevant phosphatase has not yet been identified (Wang et al., 2023b). Therefore, the mechanisms by which phosphatases activate CAT remain largely unexplored. In this study, we demonstrate that OsPP1a specifically dephosphorylates CatC at Thr-292 in the peroxisome, thereby activating its enzymatic activity both *in vitro* and *in vivo* (Figures 2B and 3). The dephosphorylated CatC efficiently scavenges excess H_2_O_2_, ultimately enhancing salt tolerance in rice (Figures 3I–3L; Supplemental Figures 9A–9D). While CAT has been shown to be activated by kinases (CPK8, STRK1, and BAK1) and the calcium sensor ROD1 at the plasma membrane (Zou et al., 2015; Zhou et al., 2018; Zhang et al., 2020c; Gao et al., 2021), it is generally deactivated by phosphatases (e.g., PC1 and PP2C1) in the peroxisome (Liu et al., 2023; Bai et al., 2024). This pattern suggests that CAT activation at the plasma membrane and deactivation in the peroxisome may represent a key regulatory mechanism. Unexpectedly, our findings reveal that CatC is activated by the phosphatase OsPP1a in the peroxisome, indicating a novel regulatory mechanism in which OsPP1a functions as a molecular switch to activate CAT. Thus, the identification and functional characterization of OsPP1a provide new insights into phosphatase-mediated activation of CAT in plants.

Protein phosphorylation is a dynamic post-translational modification that enables precise temporal control of protein structure, function, subcellular localization, and degradation (Mhamdi et al., 2012; Zou et al., 2015). CAT activity is often influenced by its stability, oligomeric state, and subcellular localization (Habets and Offringa, 2014; Li et al., 2015; Wang et al., 2023b; Liu et al., 2023). In Arabidopsis*,* CAT can be transported to the nucleus either in a pathogen effector-dependent or -independent manner, thereby affecting its enzymatic activity (Al-Hajaya et al., 2022). Recently, the acyltransferase saline–alkali tolerance and blast resistance 1 (STBR1) was shown to stabilize CatA and promote H_2_O_2_ scavenging in rice (Cheng et al., 2026). We found that the phosphorylation state of CatC at Thr-292 does not affect its peroxisomal targeting or oligomeric state (Supplemental Figures 18 and 19). However, the dephospho-mimic form (CatC^T292A^) appears more stable than the phospho-mimic (CatC^T292D^) and WT forms both *in vitro* and *in planta* (Figures 4A–4F), indicating that dephosphorylation at Thr-292 enhances CatC stability. Importantly, CatC degradation is inhibited by the proteasome inhibitor MG132 (Figures 4C–4F), suggesting the involvement of the ubiquitin–26S proteasome pathway. Specifically, the E3 ubiquitin ligase APIP6 ubiquitinates CatC and promotes its degradation via the 26S proteasome pathway, ultimately enhancing blast resistance in rice (You et al., 2022). This finding implies that the ubiquitin–proteasome system plays a key role in CAT-mediated ROS homeostasis and stress responses, although its precise regulatory mechanisms remain unclear. Our results showed that all CatC variants (CatC^T292A^, CatC^T292D^, and CatC) exhibited similar stability in *APIP6*-RNAi protoplasts (Figure 4G). However, compared with CatC, less CatC^T292A^ and more CatC^T292D^ were ubiquitinated when co-expressed with APIP6 in *N. benthamiana* leaves (Figure 4H). Similar results were observed in the *catc* mutant background under salt stress (Figure 4J). Furthermore, CatC^T292A^ was more stable than CatC^T292D^ and CatC when co-expressed with APIP6 in rice plants (Supplemental Figures 11A and 11B). These results demonstrate that dephosphorylation of CatC at Thr-292 inhibits its APIP6-mediated ubiquitination and degradation. This conclusion is further supported by the finding that dephosphorylation at Thr-292 weakens the interaction between CatC and APIP6 (Figures 4J–4N). Collectively, these results indicate that OsPP1a-mediated dephosphorylation of CatC at Thr-292 suppresses APIP6-dependent ubiquitination and degradation, thereby stabilizing CatC in rice. These findings provide new insights into the regulatory mechanisms governing CAT ubiquitination and stability in response to stress.

### OsPP1a acts as a critical regulator of salt-stress responses and maintains the balance between salt tolerance and growth

Although activation of stress responses can significantly improve survival under adverse conditions, constitutive stress tolerance is often costly, impairing growth and environmental fitness and resulting in severe yield losses in crops (Zhang et al., 2020a; Zhao et al., 2021). Therefore, stress responses must be precisely controlled to enable rapid activation under stress while minimizing growth penalties upon stress relief. Salt stress, particularly in arable land, is often temporary and can be alleviated by sufficient rainfall or effective irrigation (Liu et al., 2023). Therefore, crops must fine-tune their responses not only to withstand stress but also to ensure timely life-cycle completion after stress relief. For example, the Na^+^/H^+^ antiporter SOS1 is phosphorylated and activated by the kinase SOS2 under salt stress, enhancing salt tolerance in Arabidopsis; upon stress relief, SOS1 is dephosphorylated and inactivated by the phosphatases PP2C.D6 and PP2C.D7 to promote the transition from salt tolerance to growth and development (Fu et al., 2022). Similarly, we previously found that CatC is phosphorylated and activated by the kinase STRK1 during salt stress to enhance rice salt tolerance but is subsequently dephosphorylated and inactivated by the phosphatase PC1 upon stress relief, thereby promoting growth and development (Liu et al., 2023). These examples underscore the importance of dynamically resetting the balance between salt tolerance and growth when engineering salt-tolerant, high yield crops. In this study, we found that OsPP1a activity was significantly upregulated after salt treatment, leading to decreased phospho-threonine levels of CATs and increased CAT activity, thereby enhancing salt tolerance (Figures 5C–5E). By contrast, the activities of OsPP1a and CATs were rapidly suppressed upon stress relief. These results indicate that activation of OsPP1a and its mediated dephosphorylation of CATs are required for initiating salt-stress responses, whereas inhibition of OsPP1a activity and phosphorylation of CATs at Thr-292 are necessary for the transition from salt tolerance to growth. However, the kinase responsible for phosphorylating Thr-292 in CATs remains to be identified. We also observed that the phospho-threonine levels of CATs are significantly reduced in *OsPP1a*-overexpressing seedlings during the early stage of salt stress (within 30 min) (Figures 5F and 5G), suggesting that OsPP1a participates in early salt-stress signaling. Furthermore, seminal root growth analyses during the transition from salt stress to normal conditions revealed that *OsPP1a* overexpression promotes, whereas OsPP1a deficiency inhibits, seminal root growth under salt stress (Figures 5H–5L). In contrast, the opposite trend was observed after stress removal. Notably, in the OsPP1a-deficient background, CatC^T292A^ promotes root growth under salt stress but inhibits growth upon stress relief (Figures 5H–5L), suggesting that the non-phosphorylatable CatC^T292A^ variant can promote root growth under stress even when upstream dephosphorylation is impaired. Collectively, these findings demonstrate that OsPP1a-mediated dephosphorylation of CatC at Thr-292 represents a precise and efficient mechanism for balancing salt tolerance and growth in rice. Notably, Thr-292 is highly conserved within the CAT family (Supplemental Figure 8E), indicating that reversible phosphorylation at this residue is a key regulatory mechanism underlying the trade-off between salt tolerance and growth in plants.

**Figure 7:**
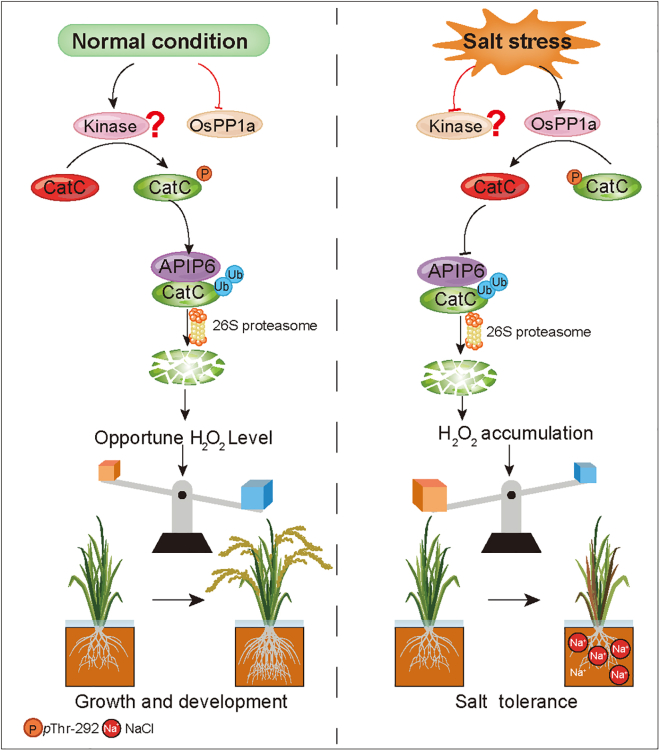
Proposed model for the role of OsPP1a in regulating salt-stress tolerance. Salt stress induces H_2_O_2_ accumulation, leading to oxidative damage in cells. Under salt stress, the phosphatase OsPP1a is activated, whereas an unknown kinase responsible for phosphorylating CatC at Thr-292 is inhibited. Activated OsPP1a then dephosphorylates CatC at Thr-292 in the peroxisome. This modification inhibits the interaction between CatC and the E3 ubiquitin ligase APIP6, thereby preventing CatC degradation via ubiquitination. The resulting stabilization of CatC enhances its capacity to eliminate excess H_2_O_2_, ultimately improving salt tolerance in rice. When salt stress is alleviated, OsPP1a activity is suppressed, while the unknown kinase becomes activated. The activated kinase phosphorylates CatC at Thr-292, promoting its degradation through the APIP6-mediated ubiquitin–26S proteasome pathway. This regulated turnover maintains intracellular H_2_O_2_ at an appropriate level, where it primarily functions as a signaling molecule, thereby supporting normal growth and development in rice. The question mark denotes the unidentified kinase. The yellow and pink shapes representing the kinase and OsPP1a indicate inactive and active states, respectively. The yellow-green and red shapes representing CatC indicate inactive and active forms, respectively.

ROS function as ancient signaling molecules that regulate numerous processes in living organisms, but they also become toxic byproducts of aerobic metabolism when excessively accumulated (Suzuki et al., 2012; Wang et al., 2024a, 2024b). Consequently, ROS homeostasis is tightly regulated. We found that, despite a marked increase in CAT activity under salt stress, H_2_O_2_ levels still rose slightly in rice seedlings (Figures 5D and 5E). This observation suggests that a transient ROS burst or moderate H_2_O_2_ accumulation is required to initiate the salt-stress response, while its levels must be maintained within a specific range to sustain salt tolerance. Additionally, both the transcript abundance and enzymatic activity of OsPP1a are induced by NaCl treatment (Supplemental Figure 2H; Figure 5C), indicating that upregulation of OsPP1a at both the transcriptional and functional levels is necessary for rice adaptation to salt stress. Given that OsPP1a activity increases sharply within the first day of salt treatment (Figure 5C) and rapidly dephosphorylates CATs (within 30 min) (Figure 5F), the early salt-stress response is likely driven primarily by enzymatic activity rather than changes in transcript abundance. Based on these findings, we propose a working model for OsPP1a (Figure 7). Upon salt stress, OsPP1a is activated, whereas the kinase responsible for phosphorylating CatC at Thr-292 is inhibited. Activated OsPP1a dephosphorylates CatC at Thr-292 in the peroxisome, thereby inhibiting its interaction with the E3 ubiquitin ligase APIP6 and preventing ubiquitination-mediated degradation. The resulting stabilization of CatC enhances its H_2_O_2_-scavenging capacity, improving salt and oxidative stress tolerance in rice. When salt stress is alleviated, OsPP1a activity is suppressed. Concurrently, the unidentified CatC kinase is activated and phosphorylates CatC at Thr-292, promoting its degradation via the APIP6-mediated ubiquitin–26S proteasome pathway. This regulated turnover maintains appropriate intracellular H_2_O_2_ levels, allowing H₂O₂ to function as a signaling molecule that supports normal growth and development. In contrast to the phosphatase PC1, previously identified as a negative regulator of salt tolerance in rice (Liu et al., 2023), OsPP1a functions as a positive molecular switch that enhances salt tolerance by stabilizing CatC and maintaining H_2_O_2_ homeostasis. The kinase responsible for phosphorylating CatC at Thr-292 remains unknown and warrants further investigation. Notably, OsPP1a has recently been reported to dephosphorylate and inactivate the kinases SAPK8/9/10, thereby negatively regulating ABA signaling; moreover, it can be oxidized and inhibited by H_2_O_2_ produced by respiratory burst oxidase homologs (RBOHs) in rice (Qin et al., 2024). Interestingly, our results indicate that OsPP1a does not merely act passively within abiotic stress signaling pathways. Instead, it actively dephosphorylates and activates CatC to eliminate excess H_2_O_2_, thereby alleviating H_2_O_2_-mediated oxidative inhibition. Together, these findings suggest that OsPP1a plays a central role in coordinating ABA signaling and abiotic stress responses.

In summary, the results of this study demonstrate that OsPP1a maintains CatC stability and H_2_O_2_ homeostasis to regulate salt-stress responses by dephosphorylating CatC at Thr-292 in rice. The identification and functional characterization of OsPP1a provide insight into the mechanisms by which protein phosphatases activate CAT in plants. The markedly improved growth and grain yield of *OsPP1a*-overexpressing rice under salt stress further indicate that *OsPP1a* is a promising candidate gene for maintaining yield in crops exposed to saline conditions.

## Methods

### Plant materials and stress treatments

The full-length coding sequence (CDS) of *OsPP1a* was cloned into the pCAMBIA1300 (hygromycin resistance) vector to generate a C-terminal FLAG fusion construct. The *OsPP1a* promoter was separately cloned into the pCAMBIA1301 (hygromycin resistance) vector upstream of the *GUS* reporter gene, yielding the construct pCAMBIA1301–*OsPP1a*_*pro*_*:GUS* using specific primers (Supplemental Table 3). Additionally, CRISPR–Cas9-mediated gene editing was used to generate knockout mutants in rice. Target sites for *OsPP1a* and *CatC* were designed using the CRISPR–Plant Web tool (http://skl.scau.edu.cn/). The CRISPR–Cas9 vector was constructed following established protocols (Ma et al., 2015), with primers listed in Supplemental Table 3. All constructs were introduced into rice (*O. sativa* L. ssp. *japonica* cv. Kitaake) via *Agrobacterium tumefaciens-*mediated transformation as previously described (Lin et al., 2009). Transgenic plants were selected based on hygromycin resistance. The *ospp1a* and *catc* mutants were identified by Sanger sequencing of the targeted genomic regions (Supplemental Table 3).

The phospho-mimic form CatC^T292D^ and dephospho-mimic form CatC^T292A^ were generated by site-directed mutagenesis, substituting Thr-292 with Asp or Ala, respectively. The CDSs of CatC^T292D^ and CatC^T292A^ (with an N-terminal FLAG tag) were cloned into the pCAMBIA1300 vector (Supplemental Figure 8B) and the pBWA(V)–FLAG vector (bialaphos resistance; Supplemental Figure 8D) using primers listed in Supplemental Table 3. The resulting constructs were introduced into the *ospp1a-23* and *catc-21* knockout mutant backgrounds via *Agrobacterium*-mediated transformation. In parallel, the CDSs of CatC variants (with an N-terminal HIS tag) and APIP6 (with a C-terminal MYC tag) were cloned into the pDT7 binary vector (hygromycin resistance; Supplemental Figure 8F) and transformed into Nipponbare rice. Positive transgenic lines were verified by immunoblotting using the following antibodies: anti-FLAG (M20004, Abmart, China; dilution 1:5000), anti-MYC (M20019, Abmart, China; dilution 1:5000), and anti-HIS (M20020, Abmart, China; dilution 1:5000).

For subsequent experiments, positive plants from the T_2_ and higher generations were used. All WT and transgenic rice plants were grown in a greenhouse hydroponic system as described by Zhou et al. (2018) under the following conditions: 28°C, 60%–70% humidity, and a 16-h light/8-h dark photoperiod.

Salt, MV, and H_2_O_2_ stress treatments of T_2_-generation transgenic and WT plants were performed as previously described (Zhou et al., 2018).

For salt stress at the seedling stage, 40 15-day-old seedlings per genotype were transferred to hydroponic solution (composition: 0.3 mM KH_2_PO_4_, 0.35 mM K_2_SO_4_, 1 mM MgSO_4_·7H_2_O, 0.5 mM Na_2_SiO_3_·9H_2_O, 1 mM CaCl_2_·2H_2_O, 9 μM MnCl_2_·4H_2_O, 20 μM H_3_BO_3,_ 0.77 μM ZnSO_4_·7H_2_O, 0.32 μM CuSO_4_·5H_2_O, 20 μM NaFeEDTA, and 0.39 μM Na_2_MoO_4_·2H_2_O, pH 5.5) supplemented with 140 mM NaCl for 10 days (Ren et al., 2005). After 8 days of recovery in normal hydroponic solution, survival rates were quantified. For enzymatic assays, leaves from identically treated seedlings exposed to 100 mM NaCl for 0, 1, 3, or 5 days were harvested for CAT activity and H_2_O_2_ concentration measurements.

For salt stress at the reproductive stage, two positive transgenic lines and WT plants (24 plants per genotype) were grown in plastic pots and subjected to 1% (w/v; approximately 170 mM) NaCl for 26 days at the panicle development stage (approximately 45 days old). The NaCl solution was then completely removed, and plants were allowed to recover under normal irrigation for 10 days before harvest. Agronomic parameters, including panicle number, weight per panicle, seed-setting rate, thousand-seed weight, and grain yield per plant, were subsequently recorded.

For MV treatment, germinated seeds (30 seeds per genotype) were transferred to hydroponic solution containing 4 μM MV. After 6 days of treatment, CAT activity, H_2_O_2_ content in leaves, and seedling height were measured.

For H_2_O_2_ treatment, three-leaf-stage seedlings (30 plants per genotype) were immersed in 100 mM H_2_O_2_ solution for 1 day. Leaves were then stained with 3,3′-diaminobenzidine solution for 1 day, decolorized in 95% ethanol for 2 h, and imaged (Noctor et al., 2016).

Seminal root growth analysis at the germination stage was performed as previously described (Liu et al., 2023). Briefly, surface-sterilized seeds (70% ethanol for 1 min, followed by 10% NaClO for 30 min) were thoroughly rinsed and soaked in sterile water at 25°C for 24 h. Germinated seeds (30°C, 24 h) were then transferred onto plastic screens floating on sterile water with or without 140 mM NaCl and incubated at 28°C. After 4 days, the NaCl solution was replaced with sterile water, and the seedlings were grown for an additional 4 days. Seminal root lengths were measured daily over an 8-day period with imaging performed every two days. Growth rates were subsequently calculated under both salt-stress and normal conditions.

### Physiological measurements

To assay CAT activity, samples were obtained from prokaryotic systems, yeast, and plant tissues following established protocols (Wang et al., 2023b). Briefly, phospho-mimic and dephospho-mimic CatC proteins were affinity-purified from *E. coli* using glutathione Sepharose 4B (GE Healthcare, UK). Similarly, *Δcta1* yeast mutant strains expressing *CatC* variants were harvested by centrifugation, and crude proteins were extracted using a one-step yeast active protein extraction kit (Sangon Biotech, China) according to the manufacturer’s instructions. Additionally, CatC variants were immunoprecipitated *in planta using* anti-FLAG beads (Sigma-Aldrich, USA). CAT activity was quantified using a commercial assay kit (product no. S0051, Beyotime, China) for immunoprecipitates, purified recombinant proteins, and yeast extracts. For soluble proteins extracted from rice, CAT activity was determined by monitoring H_2_O_2_ decomposition kinetics. Specifically, 10 μl of enzyme homogenate (10% w/v) was added to a reaction mixture containing 50 mM phosphate buffer (pH 7.0) and 10 mM H_2_O_2_ to a final volume of 2.0 mL, and H_2_O_2_ consumption was monitored at 240 nm.

H_2_O_2_ concentrations were primarily determined using a commercial kit (product no. S0038, Beyotime, China) according to the manufacturer’s instructions, with slight modifications based on Noctor et al. (2016).

Total chlorophyll content was measured as previously described with slight modifications (Ouyang et al., 2010). Briefly, total chlorophyll was extracted from 150 mg of leaf tissue using 80% acetone and quantified spectrophotometrically at 665 and 649 nm. MDA content was determined as previously described with minor modifications (Ouyang et al., 2010). Rice leaf samples (0.5 g) were homogenized in 5 ml of 10% (w/v) trichloroacetic acid and centrifuged at 5000 *g* for 10 min at 4°C. The supernatant (2 ml) was then mixed with an equal volume of 0.6% (w/v) thiobarbituric acid (prepared in 10% [w/v] trichloroacetic acid), incubated at 95°C for 15 min, and centrifuged again at 12,000 *g* for 10 min at 4°C. Absorbance was measured at 450, 532, and 600 nm. MDA content was calculated using an extinction coefficient of 155 nmol l⁻¹ cm⁻¹ and expressed as μmol g^−1^ fresh weight.

Relative ion leakage and Na^+^/K^+^ accumulation were measured as previously described (Ouyang et al., 2007; Schmidt et al., 2013).

Each data point represents the mean of three replicates. All experiments were performed three times with consistent results.

### Subcellular localization and promoter-GUS analysis

The *OsPP1a* CDS was cloned in-frame into pUC1390-GFP to generate a C-terminal GFP fusion using primers listed in Supplemental Table 3. Similarly, WT (intact) and mutant CatC variants generated by site-directed mutagenesis were cloned into the pA7-GFP vector to generate N-terminal GFP fusions. The resulting constructs were co-expressed in rice protoplasts with a peroxisome marker (CFP-PTS1) via PEG-mediated transfection (Lv et al., 2014). Subcellular localization was visualized using a confocal laser scanning microscope (Olympus FV1000, Japan) with the following excitation/emission settings: CFP (405/461–502 nm) and GFP (488/505–550 nm). Line-scan analysis of relative fluorescence intensity was performed using Fiji software by measuring pixel intensity along the indicated line (Hong et al., 2025).

For the promoter-GUS reporter assay, GUS staining of *OsPP1a*_*pro*_*:GUS* transgenic plants was performed as previously described (Asano et al., 2012). Briefly, samples were incubated in GUS staining buffer at 37°C overnight and then cleared in 75% ethanol to remove chlorophyll.

### RT–qPCR analysis

To analyze OsPP1a transcript dynamics under various stress conditions, three-leaf-stage rice seedlings were subjected to NaCl stress (140 mM), drought stress (20% [w/v] PEG 6000), alkali stress (75 mM mixed alkali: 62.5 mM NaHCO_3_ and 12.5 mM Na_2_CO_3_, pH 9.2–9.4), or oxidative stress (1% [v/v] H_2_O_2_). Shoots were harvested at 0, 2, 4, 6, 12, and 24 h after treatment for total RNA extraction using TRIzol reagent (Invitrogen, USA). First-strand cDNA synthesis and RT–qPCR were performed according to established protocols (Li et al., 2016), with rice *Actin* used as an endogenous control.

### Y2H assays

Y2H assays were performed to examine the interactions between OsPP1a and CATs (CatA, CatB, and CatC), as well as CatC variants (CatC^T292A^ and CatC^T292D^). Additionally, interactions between OsPP1 family members (OsPP1a, OsPP1b, OsPP1c, OsPP1d, and OsPP1e) and CatC were tested, with OsPFA-DSP2 used as a negative control. The self-interactions of CatC variants (CatC, CatC^T292A^, and CatC^T292D^) were also assessed as previously described (Zhou et al., 2018). Briefly, the cDNAs of *CATs* (including *CatC*^*T292A*^ and *CatC*^*T292D*^), *OsPP1s* (*OsPP1a*, *OsPP1b*, *OsPP1c*, *OsPP1d* , and *OsPP1e*), and *OsPFA-DSP2* were cloned into the pGBKT7 (bait) and pGADT7 (prey) vectors, respectively (primers listed in Supplemental Table 3). The resulting construct pairs were co-transformed into the yeast strain AH109. Transformants were selected on synthetic dropout medium lacking leucine and tryptophan (SD/–Leu/–Trp). Protein–protein interactions were further confirmed by growth on SD medium lacking Leu, Trp, His, and Ade (SD/–Leu/–Trp/–His/–Ade), and β-galactosidase activity was measured according to the manufacturer’s instructions (Clontech, Japan) using o-nitrophenyl β-D-galactopyranoside (Sangon, China) as the substrate.

### BiFC assays

BiFC assays were conducted as previously described (Zhou et al., 2018). Briefly, the cDNAs of *CatC* and *OsPP1a* were cloned into pE3308 and pE3449 (primers listed in Supplemental Table 3) to generate CatC-nVenus and OsPP1a-cCFP, respectively. These constructs were co-expressed with the peroxisome marker CFP-PTS1 in rice protoplasts. Fluorescence complementation was visualized using a confocal laser scanning microscope (Olympus FV1000, Japan).

### Pull-down assays

Full-length *CatC* and *OsPP1a* cDNAs were cloned into the pET28a and pGEX-4T-1 vectors, respectively. Recombinant proteins were expressed in *E. coli* BL21 (DE3) and affinity-purified using glutathione Sepharose 4B (Thermo Scientific, USA). For pull-down assays, 10 μg of recombinant bait GST-OsPP1a (or GST as a control) was incubated with 10 μg of HIS-CatC in binding buffer (25 mM Tris, pH 8.0, 150 mM NaCl, and 0.1% [v/v] Triton X-100) containing glutathione Sepharose 4B at 4°C for 16 h with rotation. The beads were then washed five times with binding buffer, and bound proteins were eluted in 2× SDS loading buffer at 95°C for 10 min. Protein interactions were detected by immunoblotting using anti-GST (M20007, Abmart, China; dilution 1:5000) and anti-HIS antibodies.

### CoIP assays

CoIP assays were performed to validate the OsPP1a–CatC interaction *in planta*. *OsPP1a-FLAG* and *CatC-GFP* expression constructs driven by the 35S promoter were co-expressed in *N. benthamiana* leaves via *A. tumefaciens*-mediated infiltration (Feng et al., 2008). Anti-FLAG beads were used to immunoprecipitate protein complexes, and co-immunoprecipitated proteins were eluted with 2× SDS sample buffer at 95°C for 10 min. The precipitated proteins were separated by 10% SDS–PAGE and analyzed by immunoblotting using anti-FLAG (M20004, Abmart, China; dilution 1:5000) and anti-GFP (M20008, Abmart, China; dilution 1:5000) antibodies.

### LCI assays

LCI assays were performed as previously described (Mao et al., 2020). Full-length *CatC*, its phosphomimetic variants (*CatC*^*T292A*^ and *CatC*^*T292D*^), and the interacting protein gene *APIP6* were cloned into the pCAMBIA1300-cLUC and pCAMBIA1300-nLUC vectors, respectively. Equal amounts of paired constructs were co-infiltrated into *N. benthamiana* leaves via *A*. *tumefaciens*-mediated transformation. After 2–3 days of incubation, leaves were infiltrated with 1 mM D-luciferin sodium salt (Yeasen, China) and kept in the dark for 10 min. Luminescence signals were captured using a cooled CCD imaging system (Tanon 5200 Multi, China).

### Dephosphorylation assays

*In vitro* dephosphorylation assays were performed as previously described (Liu et al., 2023). CAT proteins were immunoprecipitated from WT rice seedlings using a polyclonal anti-CAT antibody that recognizes CatA, CatB, and CatC isoforms (Liu et al., 2023). Recombinant GST-OsPP1a or GST control purified from *E. coli* was then added to an *in vitro* reaction buffer designed to simulate peroxisomal conditions (50 mM HEPES [pH 7.5], 50 mM NaCl, 0.1% [v/v] Triton X-100, 1 mM DTT, and 10 mM MnCl_2_; Shen et al., 2013). The immunoprecipitated CAT proteins were incubated at 30°C for 90 min. Phosphorylation levels were assessed by immunoblotting using phosphorylation-specific antibodies: anti-*p*Ser (ab9332, Abcam, UK; dilution 1:1000), anti-*p*Thr (ab9337, Abcam, UK; dilution 1:1000), and anti-*p*Tyr (ab17302, Abcam, UK; dilution 1:1000).

Semi-*in vitro* dephosphorylation assays were conducted as previously described with slight modifications (Liu et al., 2023). Total proteins were extracted from *ospp1a-23* mutant seedlings in 1× kinase/phosphatase buffer (25 mM Tris–HCl, pH 7.5, 50 mM NaCl, 1 mM DTT, 0.1% [v/v] Triton X-100, 5 mM MgCl_2_, 5 mM MnCl_2_, and 1 mM PMSF) supplemented with 1× protease inhibitors. Reaction mixtures containing 10 μg recombinant HIS-CatC, 25 μg total protein extract, and varying amounts of recombinant GST-OsPP1a (GST as a control) were incubated at 30°C for 90 min. Phosphorylation levels of CatC were analyzed by immunoblotting using an anti-*p*Thr antibody.

*In vivo* dephosphorylation assays were performed as previously described (Wang et al., 2015; Liu et al., 2023). WT, *OsPP1a-14*, and *ospp1a-23* rice seedlings were subjected to 140 mM NaCl stress for the indicated durations and then ground in liquid nitrogen. Total proteins were extracted, and CAT proteins were immunoprecipitated using an anti-CAT antibody conjugated to protein A/G magnetic beads (Bimake, USA). Proteins were separated by 10% SDS–PAGE, and phosphorylation levels were determined by sequential immunoblotting with anti-*p*Thr and anti-CAT antibodies.

### *In vitro* phosphatase activity assay

The phosphatase activity of OsPP1a was quantified using phosphate release assays as previously described (Liu et al., 2023). Briefly, FLAG-OsPP1a was immunoprecipitated from NaCl-treated (140 mM, various durations) *OsPP1a*-overexpressing seedlings (*OsPP1a-14*) using anti-FLAG beads (Bimake, USA). Subsequently, 10 μg of FLAG-OsPP1a was incubated with 10 nM Thr-292 phosphopeptide substrate at 30°C for 60 min in phosphatase buffer (50 mM HEPES, pH 7.4, 50 mM NaCl, 0.1% [v/v] Triton X-100, 10 mM MnCl_2_, and 1 mM DTT). Released inorganic phosphate was quantified using a malachite green phosphate assay kit (Cayman Chemical, USA), with absorbance measured at 620 nm. Phosphatase activity was expressed as pmol phosphate released·min^−1^·mg^−1^ protein.

### Protein degradation assays

To assess the effect of Thr-292 phosphorylation on CatC stability, cell-free degradation assays were performed as previously described (Liu et al., 2023; Bai et al., 2024). Total protein extracts (100 μg) were prepared from WT rice seedlings using extraction buffer (25 mM Tris–HCl, pH 7.5, 10 mM NaCl, 10 mM MgCl_2_, 10 mM PMSF, 5 mM DTT, and 10 mM ATP). These extracts were incubated separately with 10 μg of purified GST-CatC, GST-CatC^T292A^, or GST-CatC^T292D^ at 28°C for 0, 1, and 2 h. CatC degradation was analyzed by immunoblotting using an anti-GST antibody. Equal loading was verified by Ponceau S staining, and protein quantification was performed using Image Lab 4.1 (Bio-Rad, USA).

To investigate whether CatC stability is regulated by OsPP1a, total proteins were extracted from transgenic seedlings (*catc* or *ospp1a* mutants expressing *FLAG-CatC* variants) after 0, 1, 2, and 4 h of treatment with 140 mM NaCl in the presence of either 50 μM CHX or 50 μM CHX plus 10 μM MG132. Protein concentrations were determined using a BCA assay, and CatC variants were detected by immunoblotting with an anti-FLAG antibody. An anti-actin antibody (M20009, Abmart, China; dilution 1:5000) was used as a loading control.

To examine APIP6-dependent degradation of CatC, FLAG-tagged *CatC* variants (*FLAG-CatC*, *FLAG-CatC*^*T292A*^, and *FLAG-CatC*^*T292D*^) were transiently expressed in *APIP6-*RNAi protoplasts with a LUC-MYC control as previously described (You et al., 2022). *APIP6*-RNAi rice protoplasts were prepared and transformed according to Ni et al. (2019). Following transformation, protoplasts were harvested, lysed in 2× SDS loading buffer, and analyzed by immunoblotting using an anti-FLAG antibody. An anti-MYC antibody (M2002, Abmart, China; dilution 1:5000) was used to confirm equal loading.

### Ubiquitination assays

Ubiquitination assays were performed as previously described with slight modifications (Xu et al., 2021; Wang et al., 2023b). Briefly, the cDNAs of *CatC*, *CatC*^*T292A*^, and *CatC*^*T292D*^ were cloned into the pCAMBIA1300-FLAG vector, whereas *APIP6* was cloned into the pDT1–MYC vector (Mao et al., 2020). The constructs were introduced into *A. tumefaciens* strain GV3101 and co-infiltrated into *N. benthamiana* leaves in pairwise combinations. After 2–3 days post-infiltration, leaves were treated with 50 μM MG132 for 6 h. Total proteins were extracted in lysis buffer containing 50 μM MG132 and subjected to immunoaffinity purification using anti-FLAG beads at 4°C for 2 h. The beads were washed 3–4 times with lysis buffer, and bound proteins were eluted with 20 μL of 2× SDS loading buffer. Ubiquitination status was analyzed by immunoblotting using anti-ubiquitin (3936S, CST, USA; dilution 1:1000), anti-MYC, and anti-FLAG antibodies. Ponceau S staining was used to confirm equal loading.

To examine endogenous ubiquitination of CatC variants under salt stress *in planta*, *ospp1a* mutants expressing *FLAG-CatC* variants (*FLAG-CatC*, *FLAG-CatC*^*T292A*^, and *FLAG-CatC*^*T292D*^) were treated with 50 μM MG132 for 4 h, followed by 140 mM NaCl for 4 h. FLAG-tagged CatC was immunoprecipitated using anti-FLAG beads. The samples were separated by 10% SDS–PAGE, and ubiquitin conjugates were detected by immunoblotting with an anti-ubiquitin antibody. An anti-FLAG antibody was used as a loading control.

### Statistical analysis

Statistical analyses were performed using ANOVA followed by Tukey’s multiple comparisons test. Values labeled with different letters indicate statistically significant differences (*p* < 0.05).

## Funding

This work was supported by the Yuelushan Laboratory Breeding Program (YLS-2025-ZY03002, YLS-2025-ZY01004, and 2024RC2052), the 10.13039/501100001809National Natural Science Foundation of China (32372057, 32472067, 32272139, 32401768, and 32400244), the China Postdoctoral Innovative Talent Support Program (BX20230110), the Hunan Provincial Science and Technology Innovation Plan Project (2024NK1010), the Hunan Provincial Major Science and Technology Projects (20210897), the 10.13039/501100004735Natural Science Foundation of Hunan Province, China (2024JJ7239, 2021JJ40057, 2023JJ40133, 2024JJ6133, and 2021JJ30097), the Hunan Furong Program for Young Talents in Scientific and Technological Innovation(2025RC3083), the 10.13039/501100002858China Postdoctoral Science Foundation (2020M682561 and 2023M741141), the Changsha Natural Science Foundation (KQ2402061), the 2022 National Center of Technology Innovation for Saline-Alkali Tolerant Rice Functional Improvement Project (2022PT1005), the Hunan Science and Technology Innovation Plan (2025ZYJ003), and the Science and Technology Plan Project of Zengcheng District, Guangzhou (2024ZCKJ14).

## Acknowledgments

We thank Professor Yan Guo (China Agricultural University) for constructive suggestions and guidance on dephosphorylation analysis; Professor Yuese Ning (Institute of Plant Protection, Chinese Academy of Agricultural Sciences) for providing the *APIP6*-RNAi mutant rice seeds; Professor Lai-Geng Li (Shanghai Institutes for Biological Sciences, Chinese Academy of Sciences) for valuable suggestions; Professor Gao-Xing Dai (Guangxi Academy of Agricultural Sciences) for guidance on salt-stress analysis; and Professor He-Ping Zheng (The First Affiliated Hospital of Shantou University Medical College) for guidance on ubiquitination analysis and assistance in polishing the manuscript. No conflict of interest is declared.

## Author contributions

J.L. and X.L. supervised the project. Y.W., J.L., C.L., and X.L. designed the research. Y.W. and Y.T.Y. performed most of the experiments. Z.X., Z.Z., Y.T., D.M., L.Y., Z.L., S.H., and D.T. performed additional experiments. Y.N.T., X.Z., Y.Z.Y., and W.T. analyzed and evaluated agronomic traits. Y.W., C.L., and J.L. analyzed the data and wrote the manuscript.

## Supplemental information

Document S2. Supplemental Tables 1–4

Supplemental Data 1. Statistical data summary

Document S3. Article plus supplemental information
