# Supplementary material for: The protein phosphatase OsPP1a dephosphorylates and stabilizes CatC to scavenge excess H2O2 and enhance salt tolerance in rice
Source: Plant Commun. 2026 Mar 16;7(6):101824. doi: 10.1016/j.xplc.2026.101824 (PMC13261699; doi:10.1016/j.xplc.2026.101824)
Supplement: Document S1. Supplemental Figures 1–14 [file mmc1.pdf]

Plant Communications, Volume 7

Supplemental information

The protein phosphatase OsPP1a dephosphorylates and stabilizes CatC to scavenge excess H_2O_2 and enhance salt tolerance in rice

Yan Wang, YuTing Yi, ZeLin Xu, Ye Tian, DeMing Mao, ZhenDie Luo, ZhengKun Zhou, Sheng- Nan Hu, YanNing Tan, XinHui Zhao, Lei Yang, DongYing Tang, YuanZhu Yang, WenBang Tang, Cong Liu, XuanMing Liu, and JianZhong Lin

The protein phosphatase OsPP1a dephosphorylates and stabilizes CatC to scavenge excess H_2O_2 and enhance salt tolerance in rice

Short title: OsPP1a stabilizes catalase by dephosphorylation.

Yan Wang[1, 2†], YuTing Yi[1,3†], ZeLin Xu[1], Ye Tian[1, 6], DeMing Mao[2], ZhenDie Luo[1], ZhengKun Zhou[1], Sheng-Nan Hu[1], YanNing Tan[3, 5], XinHui Zhao[4], Lei Yang[1], DongYing Tang[1, 5], YuanZhu Yang[4], WenBang Tang[3, 5], Cong Liu[1, 5, 6*], XuanMing Liu[1, 5*], JianZhong Lin[1, 5, 6*]

[1] Yuelushan Laboratory, Hunan Province Key Laboratory of Plant Functional Genomics and Developmental Regulation, Hunan Research Center of the Basic Discipline for Cell Signaling, State Key Laboratory of Chemo/Biosensing and Chemometrics, Longping Agricultural College, College of Biology, Hunan University, Changsha 410082, China

[2] College of Bioscience and Biotechnology, Hunan Agricultural University, Changsha 410128, China

[3] State Key Laboratory of Hybrid Rice, Hunan Hybrid Rice Research Center, Hunan Academy of Agricultural Sciences, Changsha 410125, China

[4] Key Laboratory of Southern Rice Innovation & Improvement, Ministry of Agriculture and Rural Affairs/Hunan Engineering Laboratory of Disease and Pest Resistant Rice Breeding, Yuan Longping High-Tech Agriculture Co., Ltd, Changsha 410001, China

[5] National Center of Technology Innovation for Saline-Alkali Tolerant Rice, Changsha 410125, China

[6] Greater Bay Area Institute for Innovation, Hunan University, Guangzhou 511300, China

One-sentence summary: OsPP1a dephosphorylates CatC to promote its stability and activity by inhibiting its ubiquitination and degradation, thereby scavenging excess H_2O_2 and enhance salt tolerance salt tolerance in rice.

*corresponding Author: jianzhlin@hnu.edu.cn (J.-Z.L.), liu2022@hnu.edu.cn (C.L.), and xml05@hnu.edu.cn (X.-M.L.).

†These authors contributed equally.

Supplemental Figures and Tables

A

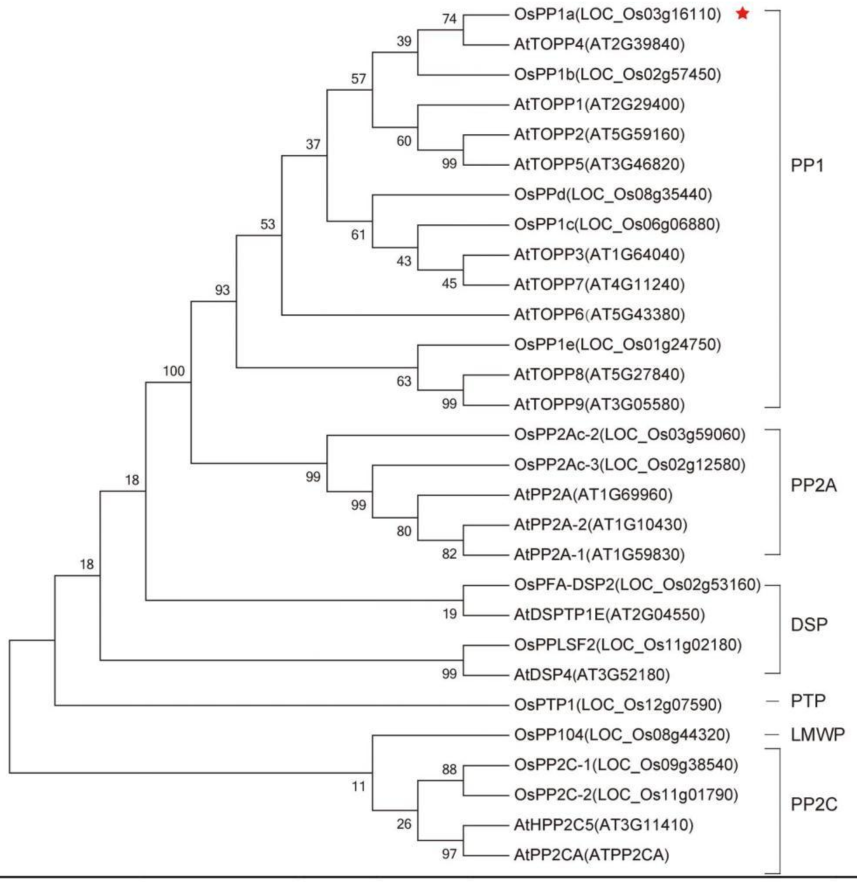

B

	OsPP1a	OsPP1b	OsPP1c	OsPP1d	OsPP1e	AtTOPP1	AtTOPP2	AtTOPP3	AtTOPP4	AtTOPP5	AtTOPP8	AtTOPP9
OsPP1a	100											
OsPP1b	79.39	100										
OsPP1c	74.40	73.91	100									
OsPP1d	55.46	57.22	59.49	100								
OsPP1e	68.28	67.28	67.47	52.21	100							
AtTOPP1	79.75	74.70	72.49	54.10	68.17	100						
AtTOPP2	82.46	75.54	75.23	56.67	67.99	81.19	100					
AtTOPP3	72.02	75.47	80.19	57.02	65.66	71.01	71.00	100				
AtTOPP4	85.19	75.99	74.03	54.25	67.88	79.63	79.81	72.84	100			
AtTOPP5	78.77	75.08	72.21	53.33	66.46	79.00	92.31	69.49	77.02	100		
AtTOPP8	70.66	67.99	69.18	52.78	71.56	67.46	70.12	67.88	69.88	69.51	100	
AtTOPP9	71.87	68.83	68.48	53.33	73.85	69.60	70.81	68.79	70.55	70.81	90.12	100

Supplemental Figure 1. Phylogenetic relationship among OsPP1a and other protein phosphatases.

(A) A phylogenetic tree of closely-related protein phosphatases to OsPP1a was constructed using MEGA 5 software with the neighbor-joining method. The full-length amino acid sequence of OsPP1a was compared with sequences in the TAIR and NCBI database. Data are presented as percentages (%). Some homologs of OsPP1a in rice and Arabidopsis were selected. Numbers indicate percentage values after 1,000 replications. On this scale, 0.02 represents a 2% change. The red pentagram indicates OsPP1a.

(B) Sequence similarity matrix of OsPP1a. Amino acid sequence identities of OsPP1a with 5 rice homologs and 7 Arabidopsis homologs are presented.

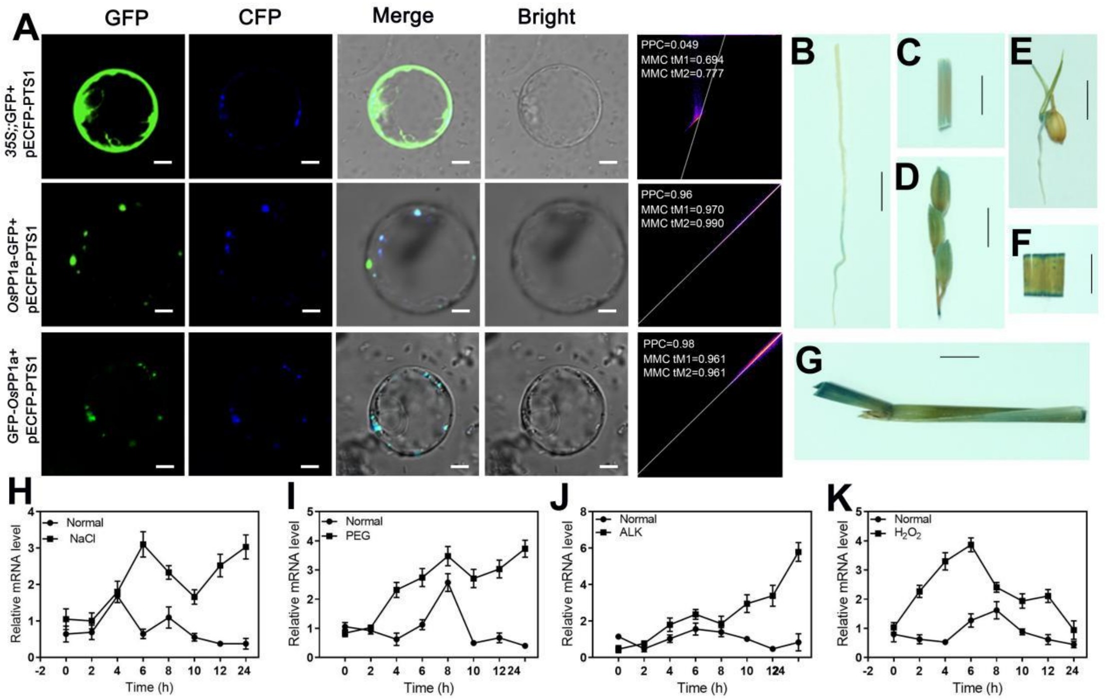

Supplemental Figure 2. Subcellular localization and expression pattern analysis of OsPP1a.

(A) Peroxisome localization of OsPP1a in rice protoplasts expressing N-terminal (GFP-OsPP1a) or C- terminal (OsPP1a-GFP) GFP fusions. *OsPP1a* CDS was amplified and cloned into pA7-GFP (N-terminal fusion) and pUC1390-GFP (C-terminal fusion). The protoplasts were isolated from 12-d-old WT seedlings. CFP-PTS1 was used to show the peroxisome. Co-localization between GFP and CFP signals was analyzed by Coloc 2 in Fiji software. PCC, Pearson's correlation coefficient; MCC, Manders' colocalization coefficient (tM1and tM2 are the proportion of colocalized signal to GFP and CFP fluorescence, respectively). The values were calculated from 20 individual images for each study. Bar = 10 μm.

(B–G) *OsPP1a* promoter-GUS expression patterns in transgenic rice. GUS expression was observed in young root **(B)**, stem **(C)**, young spikelet **(D)**, 4-d-old seedling **(E)**, leaf **(F)**, and leaf sheath **(G)**. Bar = 1 cm.

(**H–K**) Relative mRNA levels of *OsPP1a* by RT-qPCR in the three-leaf stage rice seedlings treated with 140 mM NaCl (**H**), 20% (v/v) PEG (**I**), 75 mM alkaline stress (62.5 mM $NaHCO_3$ and 12.5 mM Na_2CO_3, pH 9.2–9.4) **(J)**, and 1%(v/v) H_2O_2 **(K)**. For **(H-K)**. Data are presented as mean ± SD (n = 3).

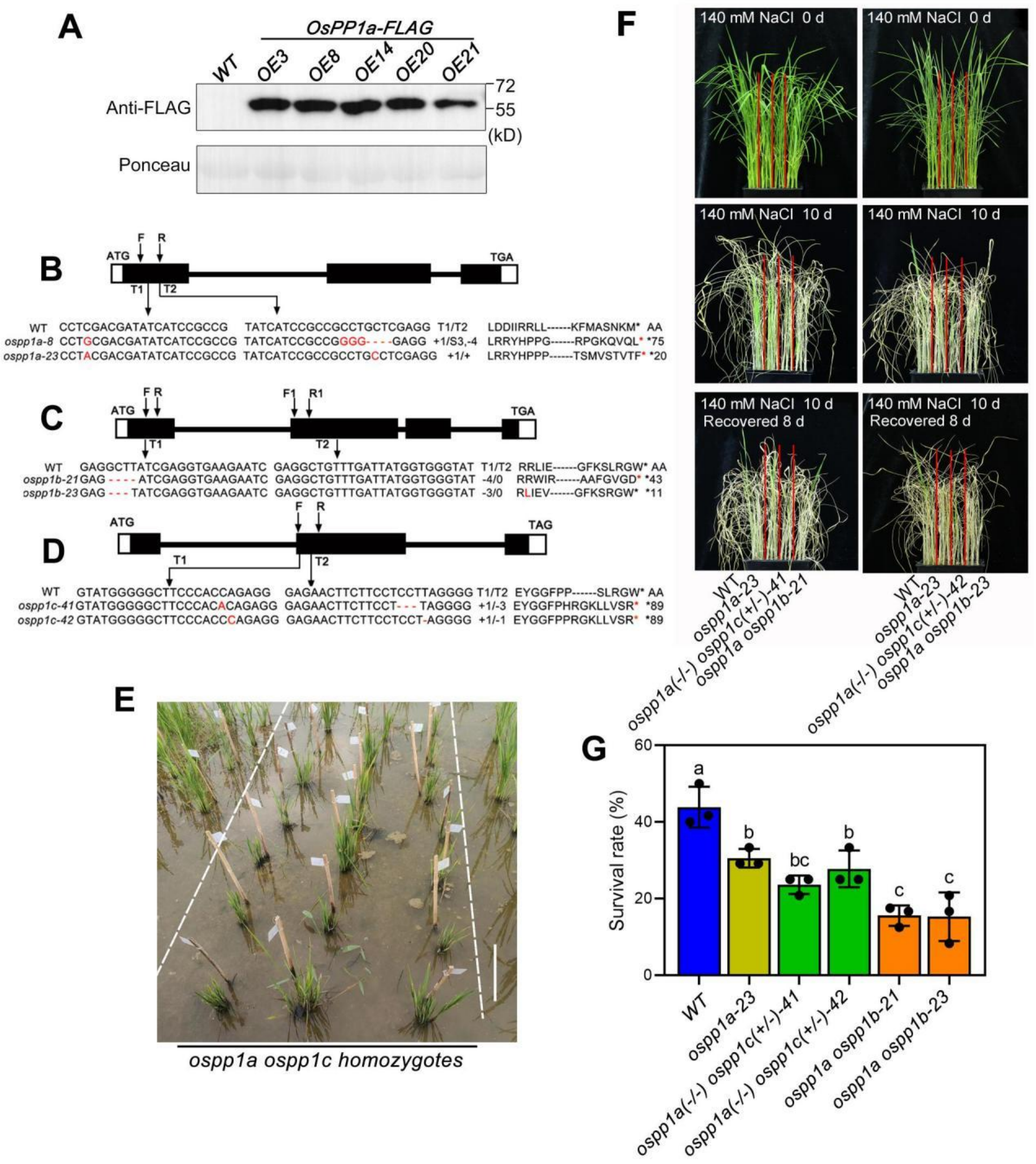

Supplemental Figure 3. Construction of *OsPP1a* overexpressing and *OsPP1s* knockout rice lines and analysis of their salt stress response.

(A) Immunoblotting analysis of *OsPP1a*-overexpressing lines and WT plants with an anti-FLAG antibody.

(B–D) Mutation details of CRISPR-edited lines *ospp1a* **(B)**, *ospp1b* **(C)**, and *ospp1c* **(D)**. The sequences of *ospp1b* and *ospp1c* lines only show the mutation details of *OsPP1b* and *OsPP1c* in *ospp1a(-/-) ospp1b(-/-)* (denoted as *ospp1a ospp1b* hereafter) and *ospp1a(-/-) ospp1c(-/-)* (denoted as *ospp1a ospp1c* hereafter) double mutants, respectively, constructed from the *ospp1a-23* background. Genomic DNA diagrams showing exons, introns, and untranslated regions are indicated by black boxes, lines between boxes, and white boxes, respectively. The sequence alignments between the mutants and WT are shown below the diagrams, with genomic sequences on the left and protein sequences on the right. Minus (－) and plus (＋) signs and the letter S indicate the

number of nucleotides deleted, inserted, and substituted, respectively, within the CRISPR/Cas9 target sequences T1 and T2. The asterisks indicate a stop codon generated. F, forward primers; R, reverse primers.

(E) The *ospp1a ospp1c* double mutants (T_0) exhibited very dwarf and sterile in the field. Bar = 10 cm.

(F and G) Photographs **(F)** and survival rates **(G)** of seedlings grown under normal conditions or salt stress (140 mM NaCl) for the indicated times. 15-d-old WT, *OsPP1a* knockout mutant (*ospp1a-23*), *ospp1a ospp1b* double mutants (*ospp1a ospp1b-21* and *ospp1a ospp1b-23*), and *ospp1a(-/-) ospp1c(+/-)* heterozygotes [*ospp1a(-/-) ospp1c(+/-)-41* and *ospp1a(-/-) ospp1c(+/-)-42*] seedlings were watered with 140 mM NaCl for 10 d and then allowed to recover for 8 d. For **(G)**, data are presented as mean ± SD. Statistically significant differences are indicated by different lowercase letters [$P < 0.05$, one-way ANOVA for **(G)** with Tukey's multiple comparisons test].

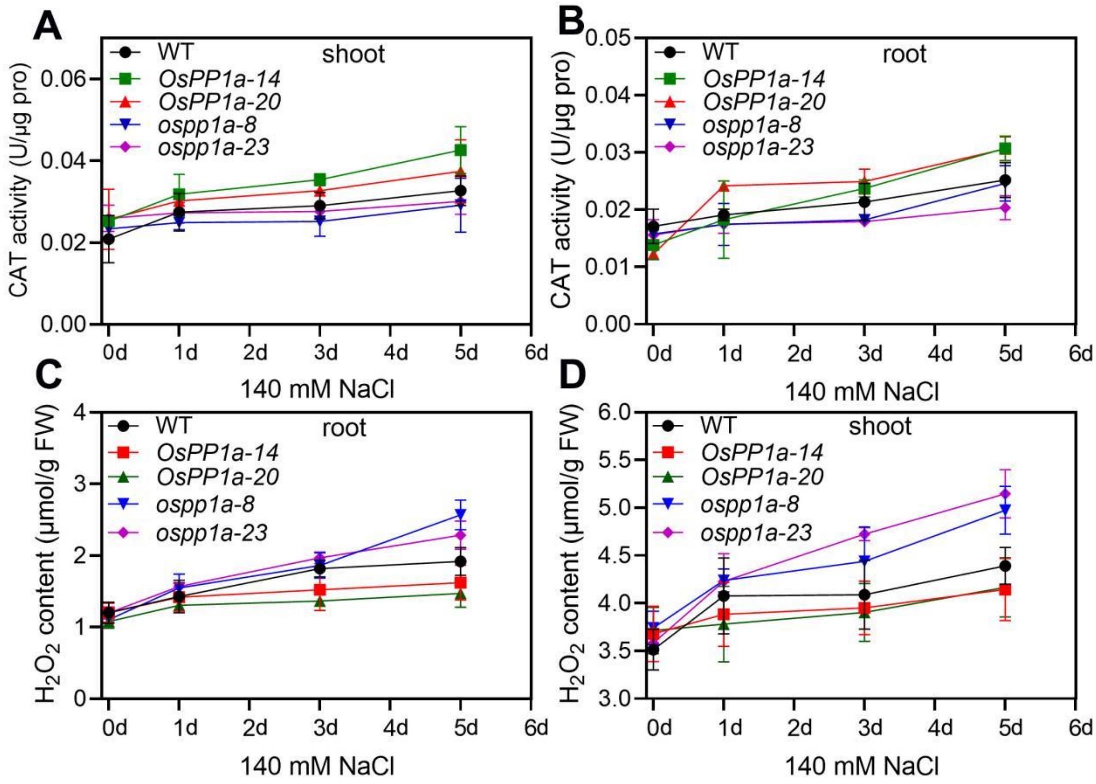

Supplemental Figure 4. OsPP1a increases CAT activity but reduces H_2O_2 accumulation in rice shoots and roots under salt stress.

(A and B) CAT activities in WT and *OsPP1a* transgenic rice shoots **(A)** and roots **(B)**.

(C and D) H_2O_2 contents in WT and *OsPP1a* transgenic rice shoots **(C)** and roots **(D)**. 15-d-old seedlings were grown under normal conditions or salt stress (140 mM NaCl) for the indicated times. For **(A-D)**, data are presented as mean ± SD (n = 3).

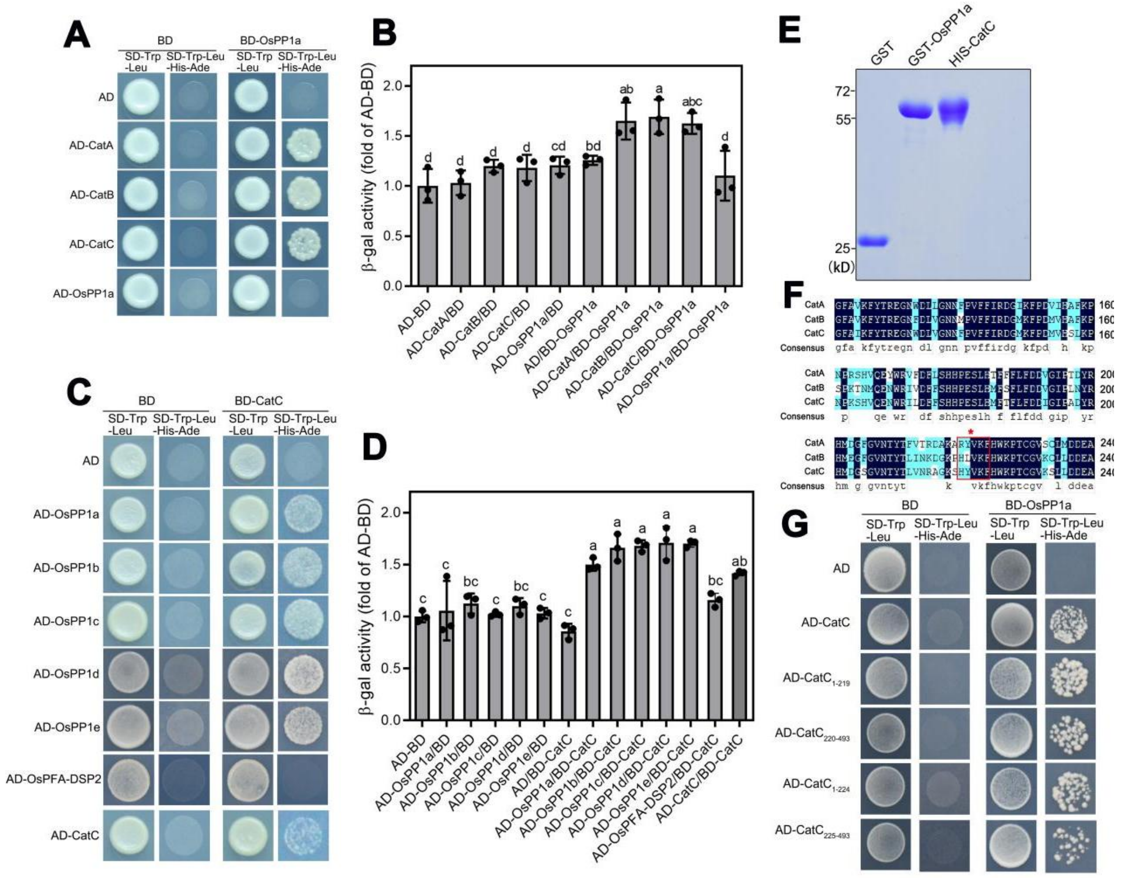

Supplemental Figure 5. OsPP1a physically interacts with CATs and CatC interacts with OsPP1s.

(A and B) Y2H **(A)** and β-galactosidase **(B)** assays of OsPP1a interaction with CATs (CatA, CatB, and CatC) and itself in yeast. The β-galactosidase activity assays quantifying the interaction in **(A)** are shown in **(B)**.

(C and D) Y2H **(C)** and β-galactosidase **(D)** assays of CatC interaction with OsPP1s (OsPP1a, OsPP1b, OsPP1c, OsPP1d, and OsPP1e), OsPFA-DSP2 (as a negative control), and itself in yeast.

(E) Coomassie-Stained SDS-PAGE Analysis of Purity for Recombinant Proteins GST-OsPP1a, GST, and His-CatC. For **(B and D)**, data are presented as mean ± SD (n = 3). Statistically significant differences are indicated by different lowercase letters ($P < 0.05$, one-way ANOVA with Tukey's multiple comparisons test).

(F) Conservation analysis of the HYVKF motif (amino acids 220-224 in CatC) in the rice CAT family. Alignment of the amino acid sequences of CatA (Os02g02400), CatB (Os06g51150), and CatC (Os03g03910) is shown, with the red box and asterisk indicating that the HYVKF motif is not conserved among the rice CAT family.

(G) Y2H assays of OsPP1a interaction with various fragments of CatC protein with or without the HYVKF motif. The fragments tested include: CatC$_{1-219}$ (N-terminal fragment lacking the HYVKF motif), CatC$_{1-224}$ (N-terminal fragment containing the HYVKF motif), CatC$_{220-493}$ (C-terminal fragment containing the HYVKF motif, and CatC$_{225-493}$ (C-terminal fragment lacking the HYVKF motif).

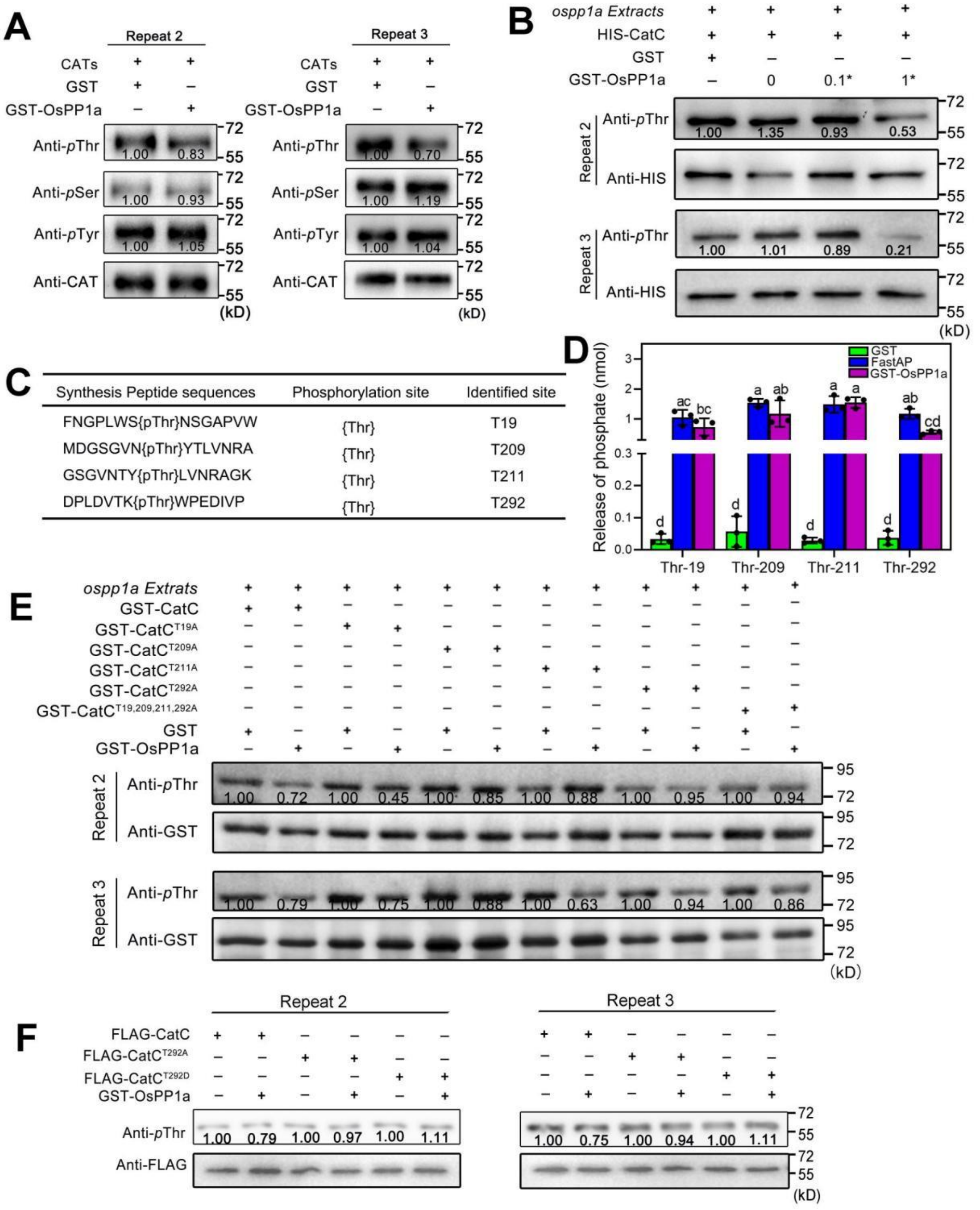

Supplemental Figure 6. Replicated experiments and additional analyses show that OsPP1a dephosphorylates CatC at Thr-292.

(A and B) The other two replicated experiments corresponding to Figure 3A and 3C further show that OsPP1a is a serine/threonine phosphatase that dephosphorylates phospho-threonines on CATs.

(C) Synthetic phosphopeptides corresponding to 4 phospho-threonine residues (Thr-19, Thr-209, Thr-211, and Thr-292) of CatC. These phosphopeptides were synthesized from Sangon (Shanghai, China).

(D) Release of phosphate from synthetic phosphopeptides by OsPP1a. In vitro phosphate measurements were performed as described by Liu et al. (2023). Thr-19, Thr-209, Thr-211, and Thr-292: synthetic phosphopeptides, corresponding to phospho-residues

Thr-19, Thr-209, Thr-211, and Thr-292 of CatC, respectively. The alkaline phosphatase FastAP and GST were used as positive and negative controls, respectively. Data are presented as mean ± SD (n= 3, **P* ≤ 0.05, ***P* ≤ 0.01, Two-way ANOVA).

(E and F) The other two replicated experiments corresponding to Figure 3D and 3H, further indicating that OsPP1a specifically dephosphorylates CatC at Thr-292. For **(D)**, data are presented as mean ± SD (n= 3). Statistically significant differences are indicated by different lowercase letters (*P* < 0.05, two-way ANOVA with Tukey's multiple comparisons test).

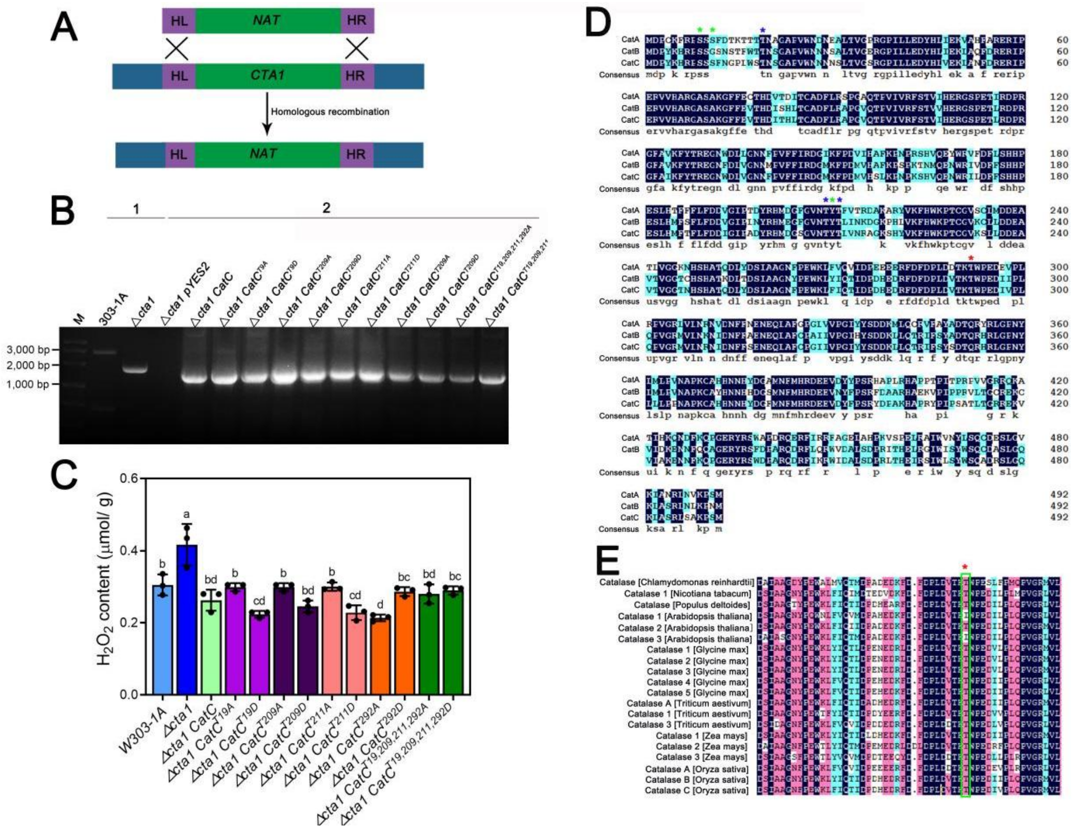

Supplemental Figure 7. Construction and identification of *⊿cta1* yeast mutant and complementary strains.

(A) Schematic diagram of *⊿cta1* yeast mutant construction. HL, the homologous primer on the left; HR, the homologous primer on the right; *CTA1*, *Cta1* gene; *NAT*, *natMX* gene. *⊿cta1* yeast is a *CAT* knockout yeast mutant, and its construction was performed as described by Wang et al. (2023c).

(B) Identification of *⊿cta1* yeast mutant and complementary strains by PCR analysis. M, DNA marker; Lane 1 products were amplified by primers CTA1A and CTA1D, and lane 2 products were amplified by primers CatC-pYES2-F and CatC-pYES2-R, shown in Supplemental Table 3. A band of about 2,176 bp was detected in *Saccharomyces cerevisiae* W303-1A (wild-type yeast), while a band of about 1,886 bp was detected in *⊿cta1* yeast mutant, suggesting that the *NAT* gene successfully replaced the *CTA1* gene. A band of about 1,500 bp is detected in Lane 2, confirming complementation of the *⊿cta1* yeast mutant by point-mutated CatC at these threonine residues.

(C) H_2O_2 contents of *⊿cta1* yeast mutant strains expressing phospho-mimic and dephospho-mimic CatCs after 2 mM H_2O_2 stress for 1 h. An anti-HIS antibody was used to detect the loading samples of CatC variants. For **(C)**, data are presented as mean ± SD (n = 3). Statistically significant differences are indicated by different lowercase letters ($P < 0.05$, one-way ANOVA with Tukey's multiple comparisons test).

(D) Sequence similarity matrix of CAT family members in rice. Alignment of the amino acid sequences of CAT family members [CatA (Os02g02400), CatB (Os06g51150), and CatC (Os03g03910)]. The green asterisks indicate the reported phosphorylation sites (Tyr-210, Zhou et al. 2018; Ser-9, Liu et al. 2023; Ser-11, Wang et al. 2023a). The blue asterisks

indicate the phospho-threonine sites we identified, and the red asterisk indicatesThr-292 in this study.

(E) Sequence conservatism of Thr-292 in the CAT family among different plants. The green box and asterisk indicate the Thr-292 in this study.

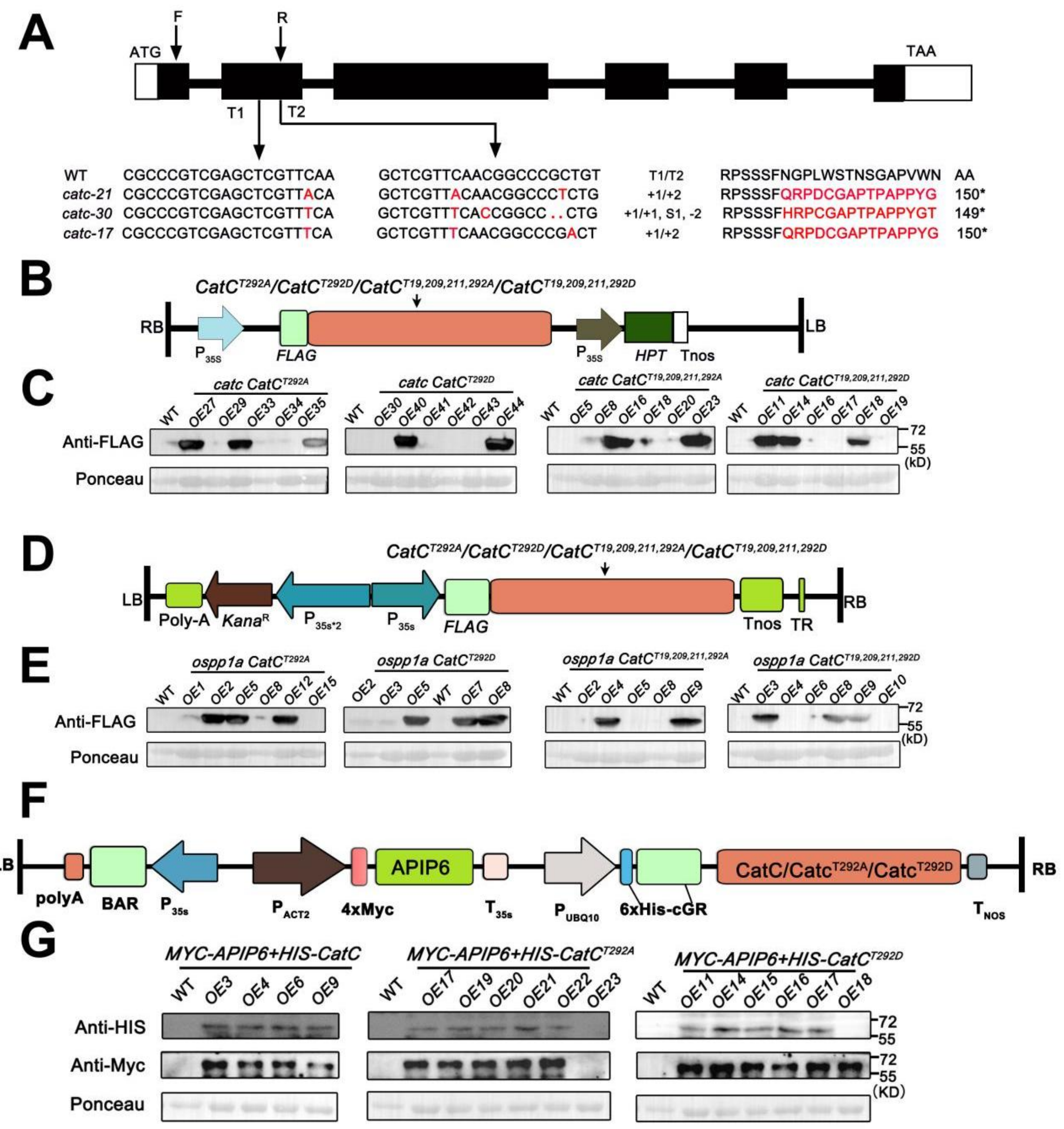

Supplemental Figure 8. Construction of transgenic plants expressing CatC variants in different background.

(A) Mutation details of CRISPR-edited lines *catc*. Diagram of the genomic DNA, with exons, introns, and untranslated regions indicated by black boxes, lines between boxes, and white boxes, respectively. Below the diagram are sequence alignments between the mutants and WT, with genomic sequences on the left and protein sequences on the right. Minus (－), plus (＋), and the letter S indicate nucleotides deleted, inserted, and substituted, respectively, within the CRISPR/Cas9 target sequences T1 and T2. The asterisk indicates a stop codon generated. F, forward primers; R, reverse primers.

(B) Schematic diagram of the plant expression vector FLAG-pCAMBIA1300 used for *CatC*^{T292A}, *CatC*^{T292D}, *CatC*^{T19,209,211,292A}, and *CatC*^{T19,209,211,292D} overexpression in the *catc* background. RB, right border; Tnos, the terminator of nopaline synthase gene (nos); *HPT*, hygromycin resistance gene; P_{35S}, CaMV 35S promoter; *FLAG*, FLAG tag gene; *CatC*^{T292A}/*CatC*^{T292D}/*CatC*^{T19,209,211,292A}/*CatC*^{T19,209,211,292D}, the CDSs of *CatC* variants; LB, left border.

(C) Immunoblotting analysis of FLAG-tagged CatC variants overexpressed in the *catc-21* mutant backgrounds using an anti-FLAG antibody. Loading was determined with Ponceau S staining (Ponceau).

(D) Diagram of the plant expression vector pBWA(V)KS-CatC used for generating $CatC^{T292A}$, $CatC^{T292D}$, $CatC^{T19,209,211,292A}$, and $CatC^{T19,209,211,292D}$ overexpression in the *ospp1a* background. $Kana^{R}$, kanamycin resistance gene.

(E) Immunoblotting analysis of FLAG-tagged CatC variants overexpressed in the *ospp1a-23* mutant backgrounds using an anti-FLAG antibody.

(F) Diagram of the plant expression pDT7 binary vector used to co-express *CatC*, $CatC^{T292A}$, and $CatC^{T292D}$ with *APIP6* in Nipponbare rice. *BAR*, bialaphos resistance gene.

(G) Immunoblotting analysis of HIS-tagged CatC variants and MYC-tagged APIP6 co-expressed in Nipponbare using anti-HIS and anti-MYC antibodies, respectively. Nip, Nipponbare rice plants. Hereafter, the transgenic plants *MYC-APIP6+HIS-CatC*, $MYC\text{-}APIP6\text{+}HIS\text{-}CatC^{T292A}$, and $MYC\text{-}APIP6\text{+}HIS\text{-}CatC^{T292D}$ were denoted as *APIP6 CatC*, $APIP6\ CatC^{T292A}$, and $APIP6\ CatC^{T292D}$, respectively.

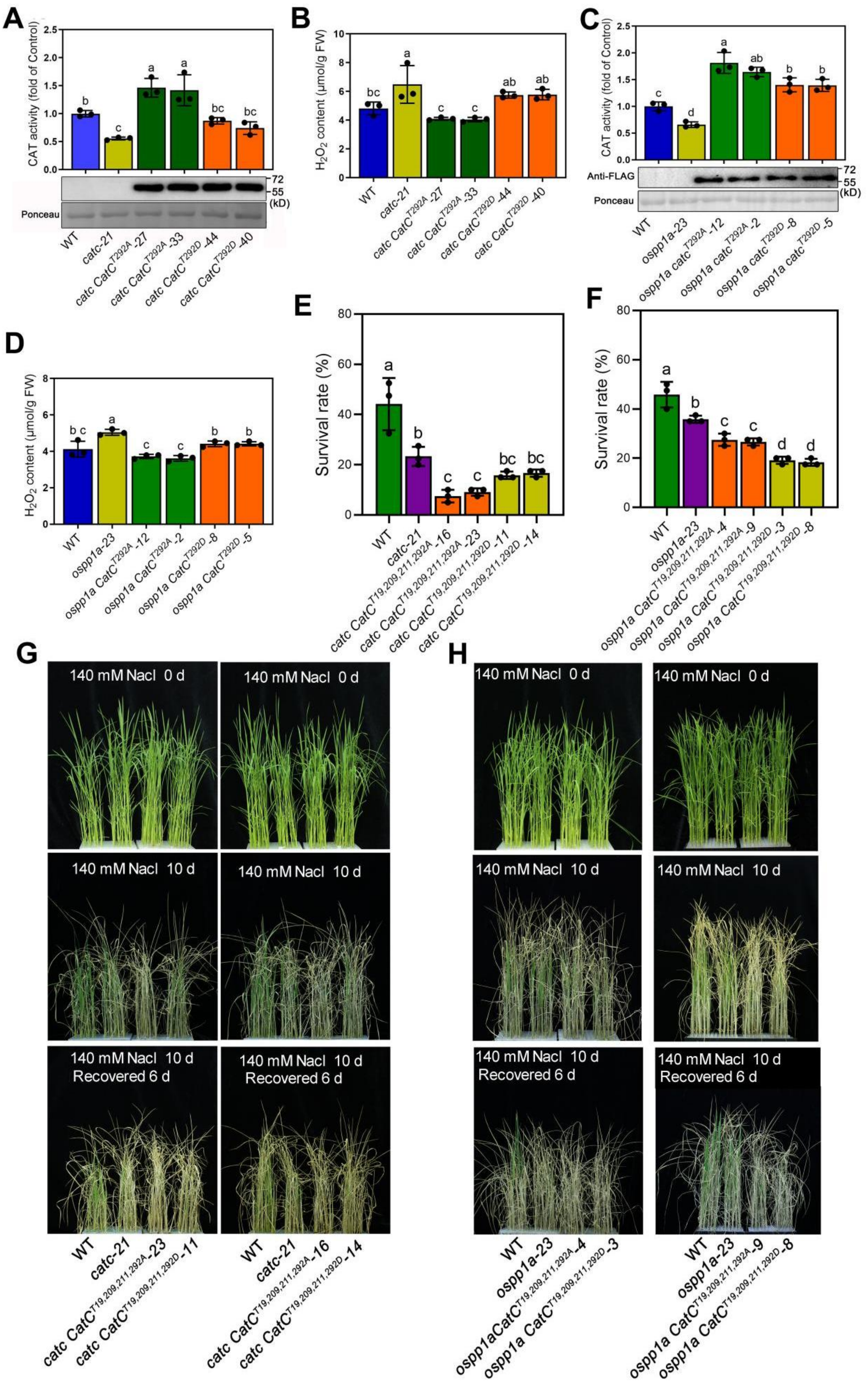

A
CAT activity (fold of Control)
0.0
0.5
1.0
1.5
2.0
2.5
b
c
a
a
bc
bc
72
55
(kD)
Ponceau
WT
catc-21
catc CatC T292A -27
catc CatC T292A -33
catc CatC T292D -44
catc CatC T292D -40
B
H2O2 content (μmol/g FW)
0
2
4
6
8
10
bc
a
c
c
ab
ab
WT
catc-21
catc CatC T292A -27
catc CatC T292A -33
catc CatC T292D -44
catc CatC T292D -40
C
CAT activity (fold of Control)
0.0
0.5
1.0
1.5
2.0
2.5
c
d
a
ab
b
b
Anti-FLAG
72
55
(kD)
Ponceau
WT
ospp1a-23
ospp1a catc T292A -12
ospp1a catc T292A -2
ospp1a catc T292D -8
ospp1a catc T292D -5
D
H2O2 content (μmol/g FW)
0
2
4
6
8
b c
a
c
c
b
b
WT
ospp1a-23
ospp1a CatC T292A -12
ospp1a CatC T292A -2
ospp1a CatC T292D -8
ospp1a CatC T292D -5
E
Survival rate (%)
0
20
40
60
80
a
b
c
c
bc
bc
WT
catc-21
catc CatC T19,209,211,292A -16
catc CatC T19,209,211,292A -23
catc CatC T19,209,211,292D -11
catc CatC T19,209,211,292D -14
F
Survival rate (%)
0
20
40
60
80
a
b
c
c
d
d
WT
ospp1a-23
ospp1a CatC T19,209,211,292A -4
ospp1a CatC T19,209,211,292A -9
ospp1a CatC T19,209,211,292D -3
ospp1a CatC T19,209,211,292D -8
G
140 mM Nacl 0 d
140 mM Nacl 0 d
140 mM Nacl 10 d
140 mM Nacl 10 d
140 mM Nacl 10 d
Recovered 6 d
140 mM Nacl 10 d
Recovered 6 d
WT
catc-21
catc CatC T19,209,211,292A -23
catc CatC T19,209,211,292D -11
WT
catc-21
catc CatC T19,209,211,292A -16
catc CatC T19,209,211,292D -14
H
140 mM Nacl 0 d
140 mM Nacl 0 d
140 mM Nacl 10 d
140 mM Nacl 10 d
140 mM Nacl 10 d
Recovered 6 d
140 mM Nacl 10 d
Recovered 6 d
WT
ospp1a-23
ospp1aCatC T19,209,211,292A -4
ospp1a CatC T19,209,211,292D -3
WT
ospp1a-23
ospp1a CatC T19,209,211,292A -9
ospp1a CatC T19,209,211,292D -8

Supplemental Figure 9. Dephosphorylation of CatC at Thr-292 increases CAT activity and reduces H_2O_2 accumulation in the *catc* and *ospp1a* mutant backgrounds.

(A and B) CAT activities **(A)** and H_2O_2 contents **(B)** in the transgenic seedlings overexpressing *CatC*^{T292A} and *CatC*^{T292D} in the *catc-21* mutant background.

(C and D) CAT activities **(C)** and H_2O_2 contents **(D)** in the transgenic seedlings overexpressing *CatC*^{T292A} and *CatC*^{T292D} in the *ospp1a-23* mutant background. WT, *catc-21*, and *ospp1a-23* seedlings were used as controls. For **(A–D)**, data are presented as mean ± SD (n = 3). Statistically significant differences are indicated by different lowercase letters ($P < 0.05$, one-way ANOVA with Tukey's multiple comparisons test).

(G and H) Photographs of seedlings grown under salt stress. 15-d-old seedlings overexpressing *CatC*^{T19,209,211,292A} or *CatC*^{T19,209,211,292D} in the *catc-21* (*catc CatC*^{T19,209,211,292A} and *catc CatC*^{T19,209,211,292D}) **(G)** or *ospp1a-23* (*ospp1a CatC*^{T19,209,211,292A} and *ospp1a CatC*^{T19,209,211,292D}) **(H)** backgrounds were treated with 140 mM NaCl for 10 d, and then allowed to recover for 6 d.

(E and F) Survival rates of transgenic lines in **(G)** and **(H),** respectively, after 6 d of recovery. 40 plants in each line were used for survival rate analysis. For **(E and F)**, data are means ± SD. Statistically significant differences are indicated by different lowercase letters ($P < 0.05$, one-way ANOVA with Tukey's multiple comparisons test).

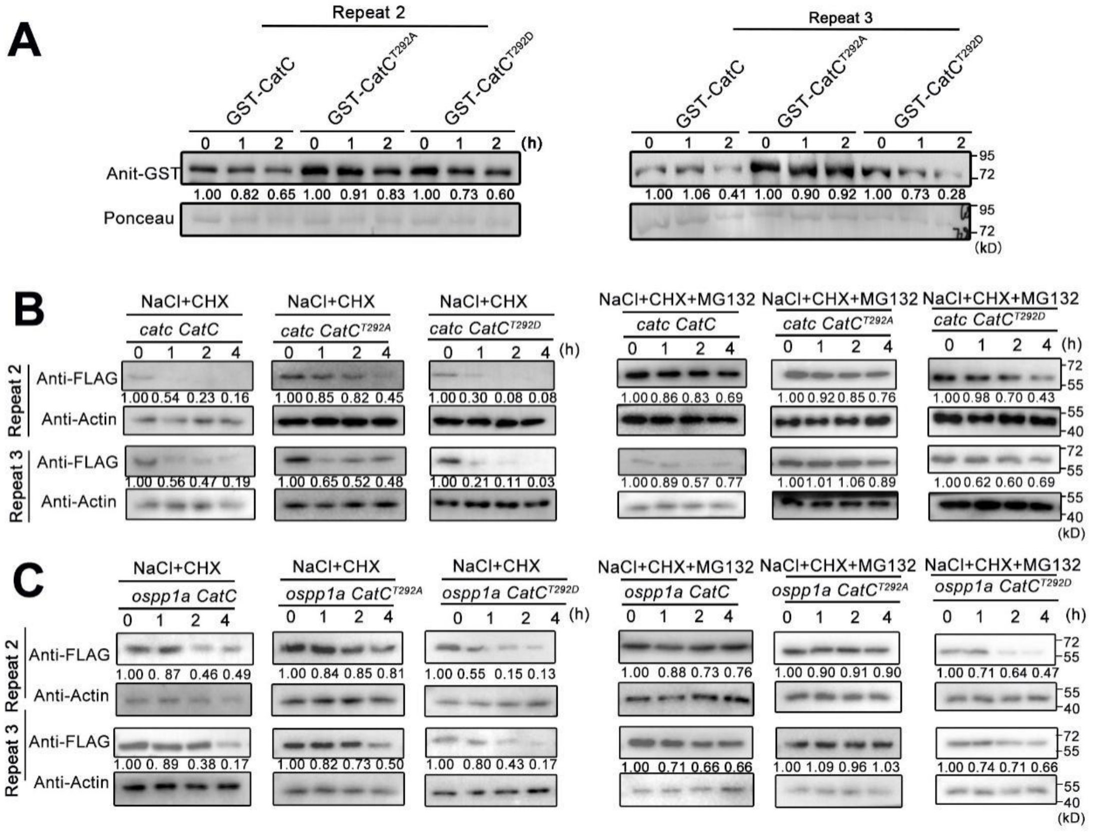

Supplemental Figure 10. The other two replicated experiments in Figure 4A, 4C and 4E.

(A–C) present the other two replicated experiments in Figure 4 A, 4C, and 4E, respectively, indicating that dephosphorylation at Thr-292 stabilizes CatC and its degradation is involved in the protein ubiquitination/26S proteasome pathway.

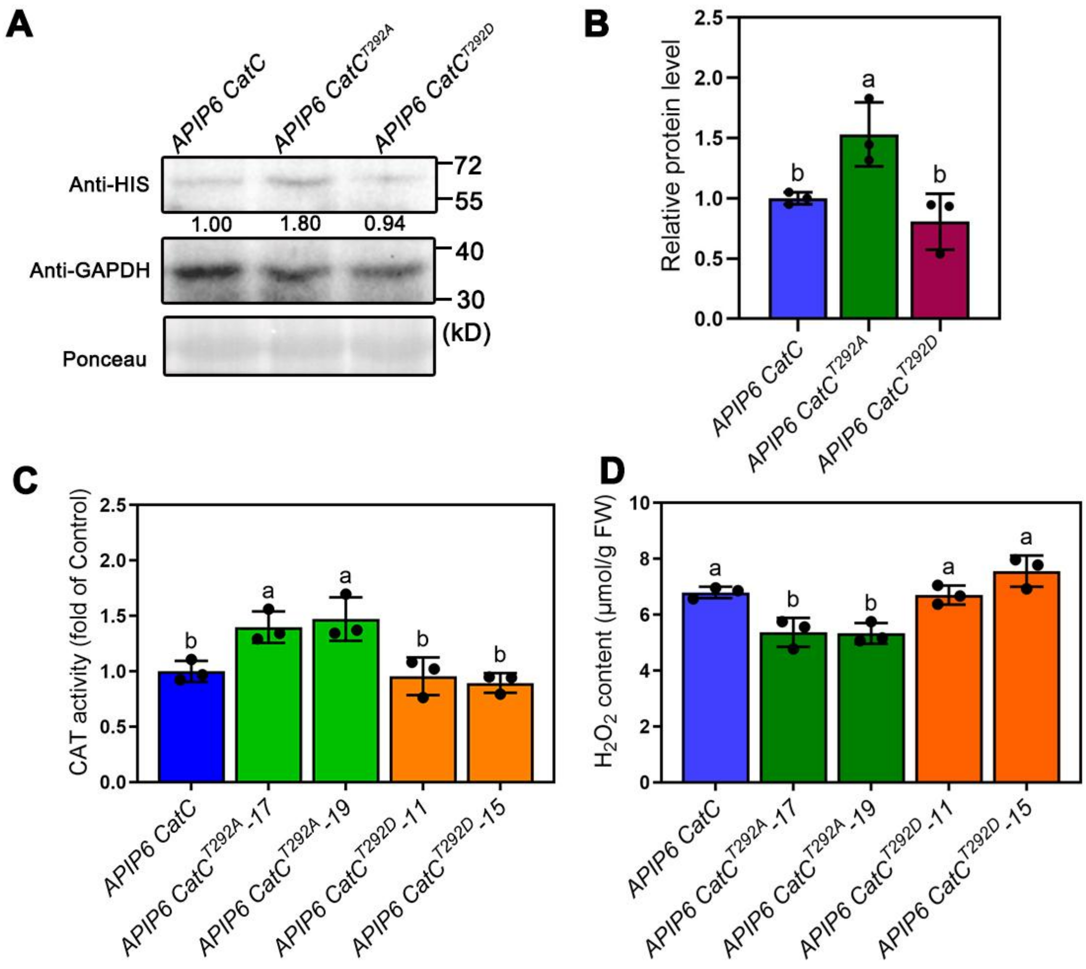

Supplemental Figure 11. Effect of Thr-292 phosphorylation on CatC protein stability and activity when co-overexpressed with APIP6 in rice plants.

(A and B) Protein stability of CatC variants **(A)** and corresponding quantification **(B)** when co-expressed with APIP6 in Nipponbare rice. Loading was determined with an anti-GAPDH antibody and Ponceau. The band intensities for anti-HIS/anti-GAPDH of *APIP6 CatC* seedlings were set to 1.

(C and D) CAT activity **(C)** and H_2O_2 content **(D)** of *APIP6 CatC*, *APIP6 CatC*^{T292A}, and *APIP6 CatC*^{T292D} seedlings under normal conditions. The CAT activity of *APIP6 CatC* seedlings was set to 1. For **(B–D)**, data are presented as mean ± SD (n = 3). Statistically significant differences are indicated by different lowercase letters ($P < 0.05$, one-way ANOVA with Tukey's multiple comparisons test).

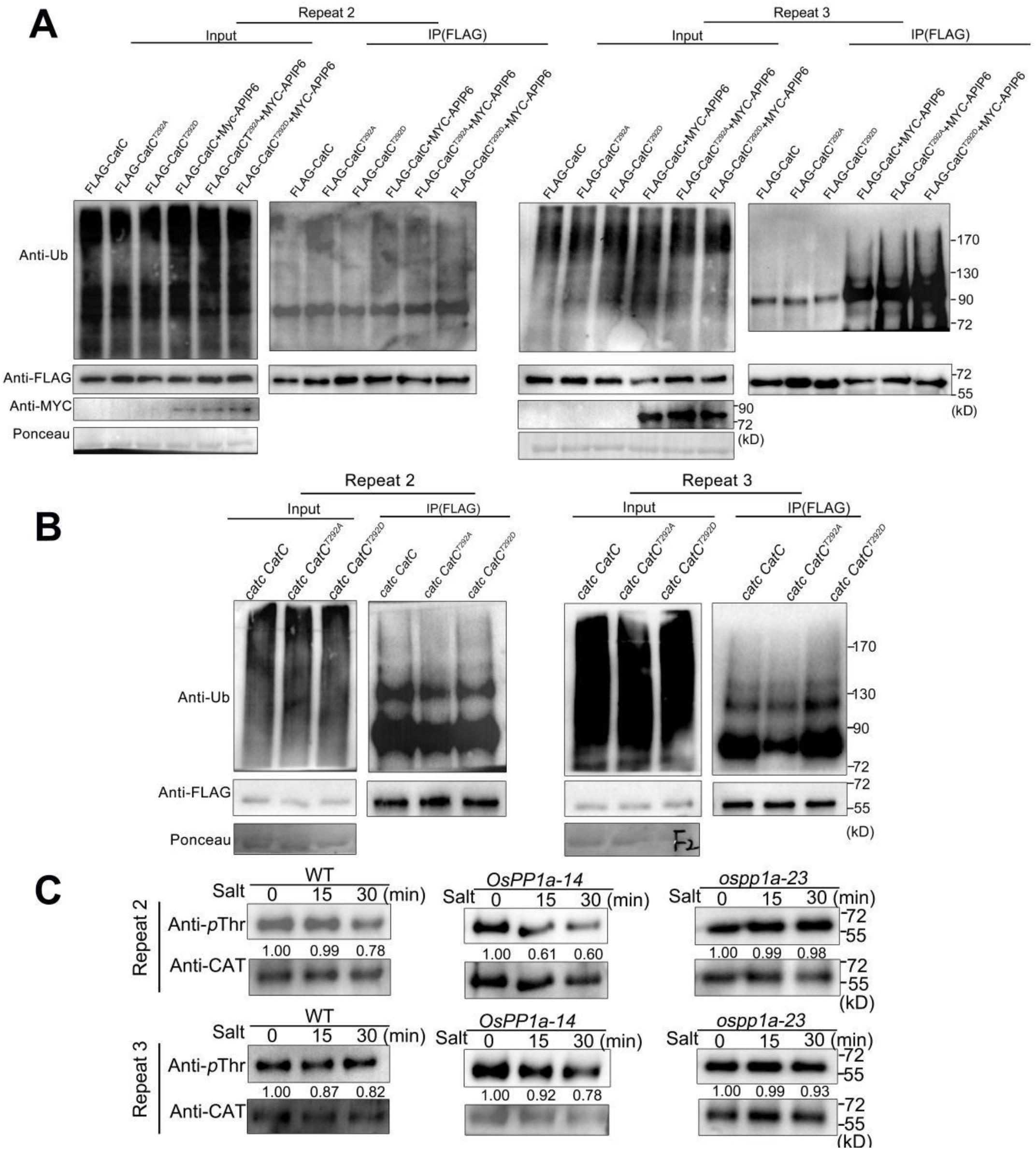

Supplemental Figure 12. **The other two replicated experiments in Figures 4 and 5.**

(A) The other two replicated experiments in Figure 4H, showing that dephosphorylation of CatC at Thr-292 inhibits its APIP6-mediated ubiquitination in *N. benthamiana*.

(B) The other two replicated experiments in Figure 4J, indicating that dephosphorylation of CatC at Thr-292 inhibits its ubiquitination in rice.

(C) The other two replicated experiments in Figure 5F showing phospho-threonine levels of CATs in WT and *OsPP1a* transgenic rice plants. The band intensities for anti-*p*Thr/anti-CAT without NaCl treatment (0 min) were set to 1.

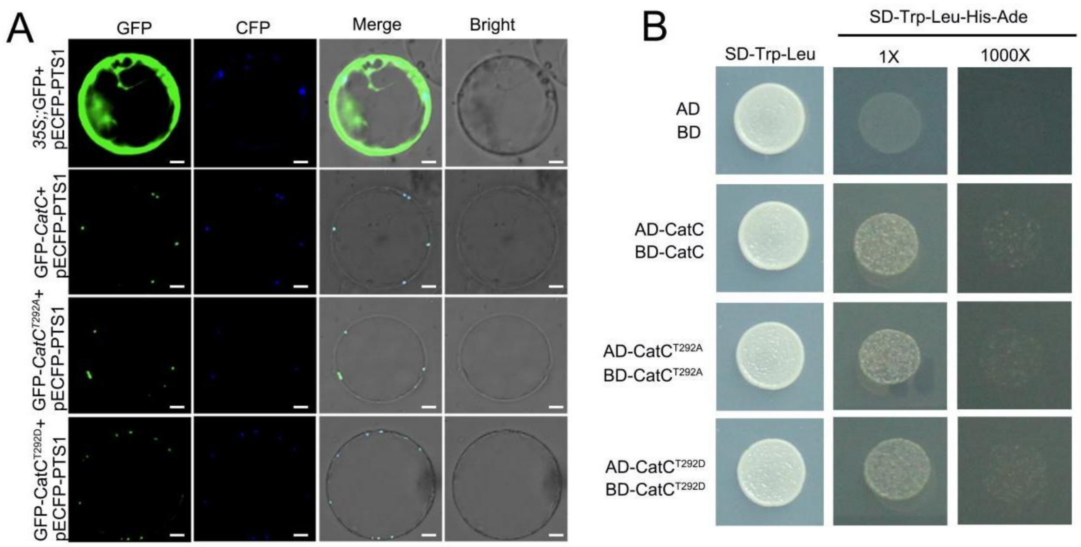

Supplemental Figure 13. Effects of Thr-292 phosphorylation on the subcellular localization and the self-interaction of CatC.

(A) Effects of Thr-292 phosphorylation on the subcellular localization of CatC. GFP was fused to the N-terminus of CatC variants, and the fluorescence was observed in rice protoplasts. The CFP-PTS1 was used to show the peroxisomes. Bar = 10 μm.

(B) Effects of Thr-292 phosphorylation on the self-interaction of CatC by Y2H assays.Y2H analysis was performed to examine the interaction between the CatC variants (CatC, $CatC^{T292A}$, and $CatC^{T292D}$) and itself, respectively. Yeast colonies were plated on SD/-Leu/-Trp medium and subsequently on selective SD/-Leu/-Trp/-His/-Ade medium. Undiluted cultures and 10^3-fold dilutions were used to assess interaction strength.

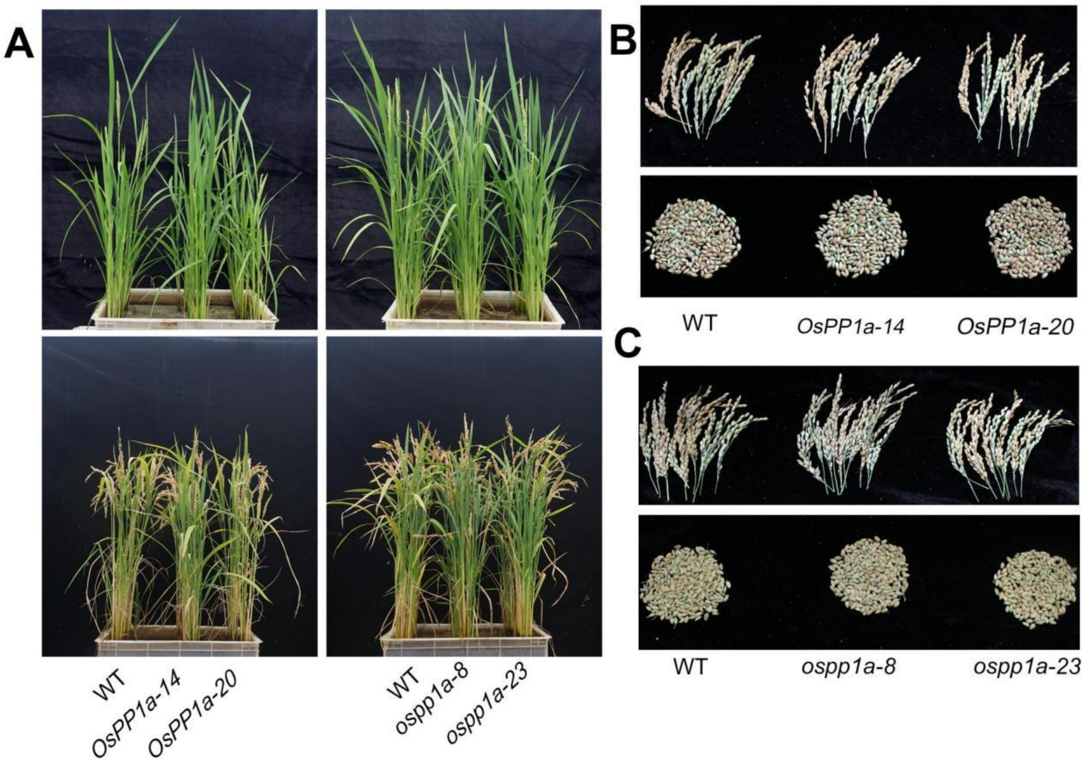

Supplemental Figure 14. No obvious difference in yield traits was observed between *OsPP1a* transgenic and WT plants under normal growth conditions.

(A) Phenotypic comparison of *OsPP1a*-overexpressing, *ospp1a mutants*, and WT plants under normal conditions at the reproductive stage. The first phenotype photos were taken at the panicle development stage (45-d-old rice plants).

(B and C) Panicles and seeds per plant of WT and *OsPP1a* transgenic rice lines under normal conditions.
